# Raman micro-spectroscopy monitors acquired resistance to targeted cancer therapy at the cellular level

**DOI:** 10.1038/s41598-018-33682-7

**Published:** 2018-10-15

**Authors:** Mohamad K. Hammoud, Hesham K. Yosef, Tatjana Lechtonen, Karim Aljakouch, Martin Schuler, Wissam Alsaidi, Ibrahim Daho, Abdelouahid Maghnouj, Stephan Hahn, Samir F. El-Mashtoly, Klaus Gerwert

**Affiliations:** 1 Department of Biophysics, Ruhr-University Bochum, 44780 Bochum, Germany; 2 Department of Molecular GI-Oncology, Clinical Research Center, Ruhr-University Bochum, 44780 Bochum, Germany

## Abstract

Monitoring the drug efficacy or resistance *in vitro* is usually carried out by measuring the response of single few proteins. However, observation of single proteins instead of an integral cell response may lead to results that are not consistent with patient’s response to a drug. We present a Raman spectroscopic method that detects the integral cell response to drugs such as tyrosine kinase inhibitors (TKIs). Non-small cell lung cancer (NSCLC) patients with EGFR mutations develop acquired resistance to first (erlotinib)- and third (osimertinib)-generation TKIs. Large erlotinib-induced differences were detected by Raman micro-spectroscopy in NSCLC cells without T790M EGFR mutation but not in cells with this mutation. Additionally, Raman difference spectra detected the response of NSCLC cells with T790M EGFR mutation to second- (neratinib) and third-generation (osimertinib) TKIs, and the resistance of cells with T790M/C797S EGFR mutation to osimertinib. Thus, the *in vitro* Raman results indicated that NSCLC cells with T790M and T790M/C797S EGFR mutations are resistant to erlotinib- and osimertinib, respectively, consistent with the observed responses of patients. This study shows the potential of Raman micro-spectroscopy to monitor drug resistance and opens a new door to *in vitro* companion diagnostics for screening personalized therapies.

## Introduction

Resistance to chemotherapy and targeted cancer therapy is one of the major obstacles in the cancer research^1^. Acquired resistance is developing during treatment of cancer patients who were initially responding to the therapy. Drug resistance is mediated by mutations acquired during therapy, in addition to other adaptive responses^2^. The currently used *in vitro* assays to monitor the drug efficacy and acquired resistance are often performed using fluorescently labelled drug molecules, Western blot, and cytotoxicity assays. Despite the high molecular specificity provided by fluorescence molecules, they are frequently much larger than the drug molecules and can significantly alter the pharmaceutical activity of the drug^3^. Western blotting monitors individual proteins and single signal transduction pathways using single proteins marked by antibodies. However, proteins are highly coupled within networks and signal transduction networks are complex. Therefore, the cellular response to drugs is most likely disturbed by crosstalk and compensation by other pathways^4–7^. Furthermore, cytotoxicity assays provide limited insights into the drug efficacy and the mechanism of drug action^8,9^. Thus, there is an unmet need for the development of a simple, non-invasive, and high throughput analytical method to evaluate the efficacy of drug candidates and predict the acquired drug resistance *in vitro* during early stages of drug discovery^3^. In addition, the results of this method should be consistent with the clinically observed responses of patients.

In recent years, Raman micro-spectroscopy has gained significant attention, especially in the analysis of biological samples. This is because biochemical information is provided by label-free Raman micro-spectroscopy with a minimum sample preparation and without using external labels or dyes^10–19^. Raman micro-spectroscopy has been used for the diagnosis of cancer using tissue biopsies and body fluids^19–28^. It is also used to image cellular components and monitor the localization of drugs in cells^29–37^. Confocal Raman microscopy can provide a read-out of the integral and physiological biochemical status of cancer cells^10–17^. For instance, Raman difference spectra of colon cancer cells before and after incubation with panitumumab antibody, which has been used in targeted colorectal cancer therapy, were recently used to monitor the impact of oncogenic K-Ras mutations on the integral cellular response^16^. Raman results were in agreement with the clinically observed patient’s response, where patients with K-Ras mutations failed to respond to panitumumab therapy. Here, we investigate the integral cellular response and resistance to targeted lung cancer therapy using small molecule tyrosine kinase inhibitors (TKIs) by Raman micro-spectroscopy.

Mutations in tyrosine kinases (TKs) and activation of their intracellular signalling transduction pathways are connected to cancer development and acquired resistance. Epidermal growth factor receptor (EGFR) is a transmembrane protein with a TK domain and is a member of the human epidermal receptor (HER) family^38^. The receptor dimerization and autophosphorylation of the intracellular TK domain is induced by the binding of ligands (EGF and TGF-α) to EGFR, stimulating cell proliferation and differentiation^39^. Since, the overexpression of EGFR stimulates tumorigenesis, angiogenesis, and metastasis, blocking of EGFR pathway is one of the major strategies for targeted cancer therapy^40–43^.

First-generation TKIs such as erlotinib (Tarceva) and gefitinib (Iressa) inhibit the phosphorylation of the TK domain of EGFR, promote apoptosis, and inhibit cell proliferation and angiogenesis. They are clinically approved by U.S. Food and Drug Administration (FDA) for the treatment of patients with activating EGFR mutation (L858R) suffering from advanced or metastatic non-small-cell lung cancer (NSCLC)^44–47^. However, acquired resistance is developed after an average of one year of the therapy. This is triggered by a second point mutation in EGFR (T790M)^48–51^, that prevents the inhibitor binding by inducing steric hindrance in the adenosine triphosphate (ATP) binding pocket^49,52,53^.

To overcome this resistance, second-generation TKIs, irreversible inhibitors that bind to Cys797 of EGFR, were developed and found to be active against the T790M resistant mutation in EGFR^54,55^. For instance, neratinib (Nerlynx) showed very promising results in clinical trials phase II for NSCLC and phase III for HER2-positive early stage breast cancer and it was approved last year for treatment of an early stage HER2-positive breast cancer^56^. As second-generation TKIs are non-selective and target both wild-type (WT) and T790M EGFR, third-generation TKIs have been developed to target only mutated EGFR^57–59^. They bind only to the mutated EGFR because their chemical structures are not built up based on the quinazoline core similar to second generation TKIs but are based on pyrimidine core which allows an up to 100-fold higher specificity for the mutated EGFR and on the same time decreases the specificity for the WT up to 100-fold^57^. They largely spare WT EGFR thus decreasing toxicity and allowing the use of doses that fully overcome T790M mutation. For instance, durable responses in T790M EGFR-mutant NSCLC patients with acquired resistance to other TKIs were induced by osimertinib (Tagrisso) that was approved recently by both FDA and European Medicines Agency (EMA) for T790M mutation-positive NSCLC^60,61^. It was also reported very recently that acquired resistance to the third generation TKIs is generated in cell lines derived from an erlotinib-resistant cancer patient^62,63^. This acquired resistance was attributed to C797S mutation of EGFR, which prevented the suppression of EGFR.

Here, we used Raman spectral imaging to monitor the integral cellular response and resistance to first-, second-, and third-generation TKIs in a label-free manner at a cellular level. The conventional *in vitro* methods in studying drug efficacy such as Western blot, cell viability assay, fluorescence microscopy, and real-time cell analysis (RTCA) were also used to characterize NSCLC cells. Raman hyperspectral images of NSCLC cells were analysed with multivariate methods such as hierarchical cluster analysis (HCA). To our knowledge, we report for the first time *in vitro* Raman spectroscopic evidence that detects the effect of EGFR mutations on the cellular response to different generations of TKIs by using cells with and without EGFR mutations. The *in vitro* Raman results are consistent with the clinically observed responses of patients. For instance, the results indicate that NCI-H1975 cells harbouring the T790M EGFR mutation are resistant to the first-generation TKI, erlotinib, but experience a strong and physiologically relevant response to the second-generation TKI, neratinib, whereas Calu-3 cells without T790M EFGR mutation experience physiologically relevant response to either erlotinib or neratinib. Furthermore, the results show that NCI-H1975 cells harbouring the T790M EGFR mutation respond to third-generation TKIs such as osimertinib and WZ4002, whereas NCI-H1975 cells harbouring the T790M/C797S EGFR mutations are resistant to these inhibitors.

## Results and Discussion

### Resistance of first- and second-generation TKIs

**Figure 1:**
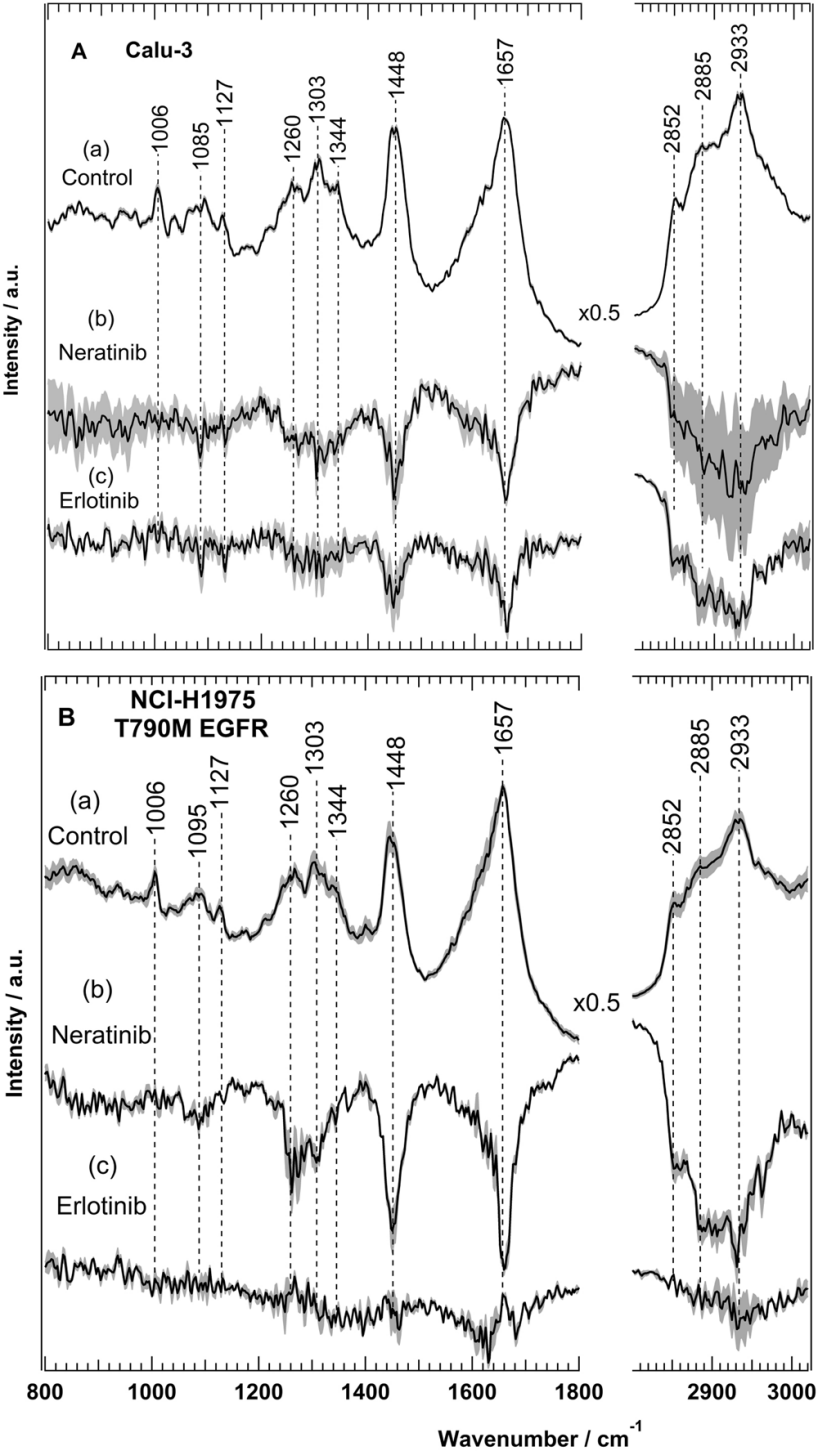
Raman difference spectra of Calu-3 (**A**) and NCI-H1975 (**B**) cells of control — cells treated with either neratinib (b) or erlotinib (c). The Raman average spectra of untreated Calu-3 and NCI-H1975 cells (a, control) are also shown in (**A**) and (**B**), respectively.

Erlotinib binds reversibly to the ATP binding site of EGFR. It blocks downstream signalling transduction and cancer progression^46^. Patients develop resistance to erlotinib after a progression-free period of about 10 months as a result of T790M EGFR mutation^48–51^. To overcome this resistance, second-generation TKIs such as neratinib were developed^64^. Neratinib forms an irreversible covalent bond with the Cys797 of EGFR and also targets HER2 and HER4^54^. Here, we used Raman spectral imaging as *in vitro* assay not only to monitor the effect of erlotinib and neratinib binding to receptors but also to predict the drug resistance at a cellular level. Several Raman spectral imaging of NSCLC cells such as Calu-3 and NCI-H1975 (approximately 60 cells per cell-line) were performed. NCI-H1975 cells harbour T790M mutation in EGFR, while Calu-3 cells don’t. Figure 1 displays the average Raman spectrum of Calu-3 cells (a, control). It contains C-H stretching modes (CH_3_, CH_2_, and CH) located near 3020–2800 cm^−1^, carbonyl stretching vibrations (amide I) located at 1657 cm^−1^, and C-H and CH_2_ bending deformation modes located at 1448 cm^−1^. The amide III vibration of peptide linkages is located near 1350–1260 cm^−1^, the band at 1127 cm^−1^ (skeletal C-C stretching), 1095 cm^−1^ (phospholipid C-C stretching) and the phenylalanine ring-breathing mode is located at 1006 cm^−1^^65,66^. To elucidate the underlying molecular changes within cancer cells upon drug treatment, Raman difference spectra (control – drug-treated cells) that reflect the overall cell response to the drug^16,17^, were calculated.

The Raman difference spectra of Calu-3 cells (control) versus cells treated with either neratinib (b) or erlotinib (c) are presented in Fig. 1A. Large spectral changes were detected, where strong negative peaks are shown near 2850–2930 (C-H stretching; lipids and proteins), 1657 (amide I; proteins), 1448 (C-H deformation; lipids), 1260–1340 (amide III; proteins and/or nucleic acids), 1127 (C-N stretching; lipids), and 1085 cm^−1^ (phospholipid C-C stretching; lipids)^67^. These spectral changes can refer to significant changes in the major cell constituents such as lipids, proteins, and nucleic acids. For instance, the changes in the amide I and amide III are associated with proteins, whereas the changes in C-H deformation and stretching, and C-C stretching originate from lipids^66,67^. The changes near 1303–1320 cm^−1^ can also originate from nucleic acids^67^. These drug-induced biochemical changes influencing subcellular components to end up in arresting the cell cycle leading subsequently to apoptosis^68^.

These results demonstrate that the inhibition of EGF receptors is reflected by changes in the Raman difference spectra. Similar to Calu-3 cells large spectral changes were observed for NCI-H1975 cells-treated with neratinib (Fig. 1B(b)). These spectral changes are free from the contribution of TKIs Raman bands (Figs S1 and S2 in SI). On the other hand, very small spectral changes were detected for NCI-H1975 cells-treated with erlotinib (Fig. 1B(c)) in contrast to Calu-3 cells (Fig. 1A(c)). These results indicate that T790M EGFR mutation in NCI-H1975 cells has a crucial role in determining the efficacy of erlotinib therapy. This is in agreement with the observed erlotinib resistance in NSCLC patients with T790M EGFR mutation^48–51^.

Furthermore, we have recently used a combination of Raman and fluorescence imaging, and cluster analysis to monitor the effect of EGFR inhibitors on subcellular components of colon cancer cells^16,17^. Similar strategy was applied in the present study to extract first the average cluster spectra of the plasma membrane region, cytoplasm, nucleus, and lipid droplets for drug-treated cells and the control. Examples of the average spectra of Calu-3 cells treated with neratinib and control are displayed in Fig. S3. Only the average spectra of nucleus contain the DNA marker band near 790 cm^−1^ (O-P-O backbone stretching). The average spectra of lipids droplets are similar to those of high lipid or phospholipids contents^16,17^. The difference spectra (untreated cells — drug-treated cells) of the plasma membrane region, cytoplasm, nucleus and lipid droplets were also calculated and shown in Figs S4–S6. These difference spectra revealed spectral changes implying variations in the subcellular organelles upon treatment with either neratinib or erlotinib. Raman difference spectra for the plasma membrane, cytoplasm, and nucleus display negative peaks, implying that the Raman intensity of these components is increased in drug-treated cells. This is most likely due to an increase in the expression level of drug-stress associated proteins upon drug treatment^69^.

**Figure 2:**
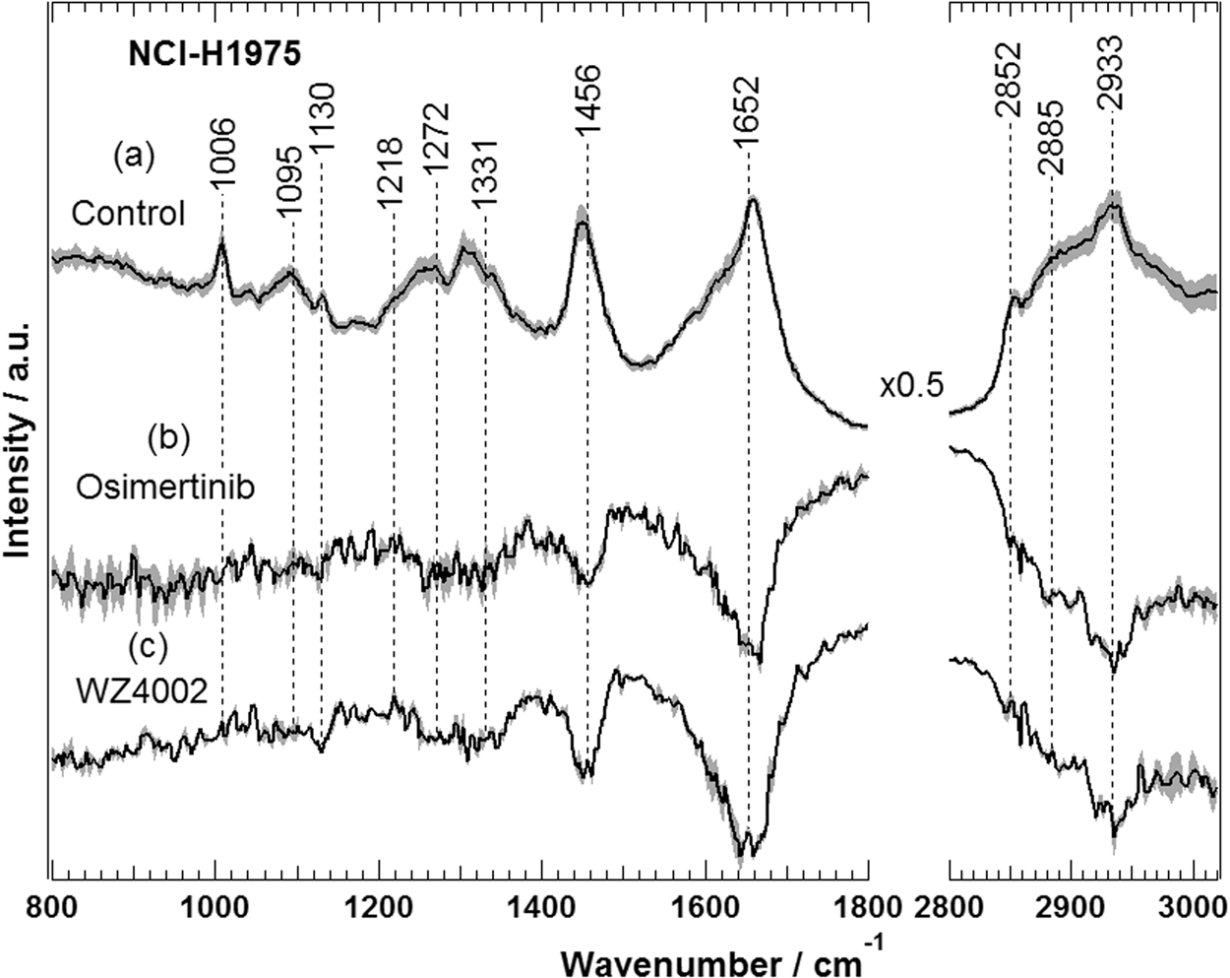
Raman difference spectra of NCI-H1975 cells of control — cells treated with either osimertinib (b) or WZ4002 (c). The Raman average spectrum of untreated NCI-H1975 cells (a) is also shown.

In the case of NSCLC patients with T790M EGFR mutation, clinical toxicity was reported due to residual activity against WT EGFR versus mutant EGFR^70^. Therefore, third generation TKIs, including osimertinib and WZ4002, have been developed to specifically target T790M EGFR mutant^57–59^. This class of inhibitors binds covalently to Cys797, and largely spares WT EGFR, thereby decreasing toxicity and permitting the use of doses that fully suppress T790M EGFR. Here, we used Raman spectral imaging to monitor the selectivity of osimertinib and WZ4002 towards mutated T790M EGFR using NCI-H1975 cells harbouring T790M EGFR. Osimertinib is approved by FDA and EMA for treatment of T790M EGFR-mutant NSCLC patients, while WZ4002 is currently in the preclinical development^71^. The Raman difference spectra of cells (control) versus cells treated with either osimertinib (b) or WZ4002 (c) are presented in Fig. 2. Large spectral changes were observed for NCI-H1975 cells treated with osimertinib (b) or WZ4002 (c), where strong negative peaks are detected near 2930–2850 (C-H stretching; lipids and proteins), 1652 (amide I; proteins), 1456 (C-H deformation; lipids), 1331–1260 (amide III; proteins and/or nucleic acids), and 1130 cm^−1^ (skeletal C-C stretching; lipids). These results suggest that the impact of the mutated T790M EGFR inhibition is revealed by changes in the Raman difference spectra. Previous studies indicated that TKIs induced G phase cell cycle arrest, and this effect might be also dependent on EGFR inhibition^72–75^. Thus, TKI-driven biochemical changes of cells would result in cell cycle arrest and eventually lead to apoptosis. Furthermore, the present Raman results (Fig. 2) are compatible with the clinical observations where patients harbouring T790M EGFR mutation responded to osimertinib^61,76^.

In order to confirm that EGFR and HER2 internalization occurs in cancer cells under the experimental conditions of the Raman measurements, cells were labelled with EGFR- and HER2-specific antibodies and fluorescence imaging was acquired. As shown in Figs S7 and S8, the majority of the EGFR and HER2 were localized within the region of the plasma membrane and in the cytoplasm. The cytoplasmic accumulation of EGFR and HER2 was enhanced in cells treated with EGF (positive control) or TKIs implying that the receptor-drug complex was internalized. These results are consistent with those reported for cells-treated with either erlotinib- or neratinib-treated cells^16,77^. It is also reported that erlotinib blocks HER2 kinase and downstream signalling events in NSCLC implying that HER2-erlotinib complex is internalized^78^, as shown in Figs S7 and S8.

**Figure 3:**
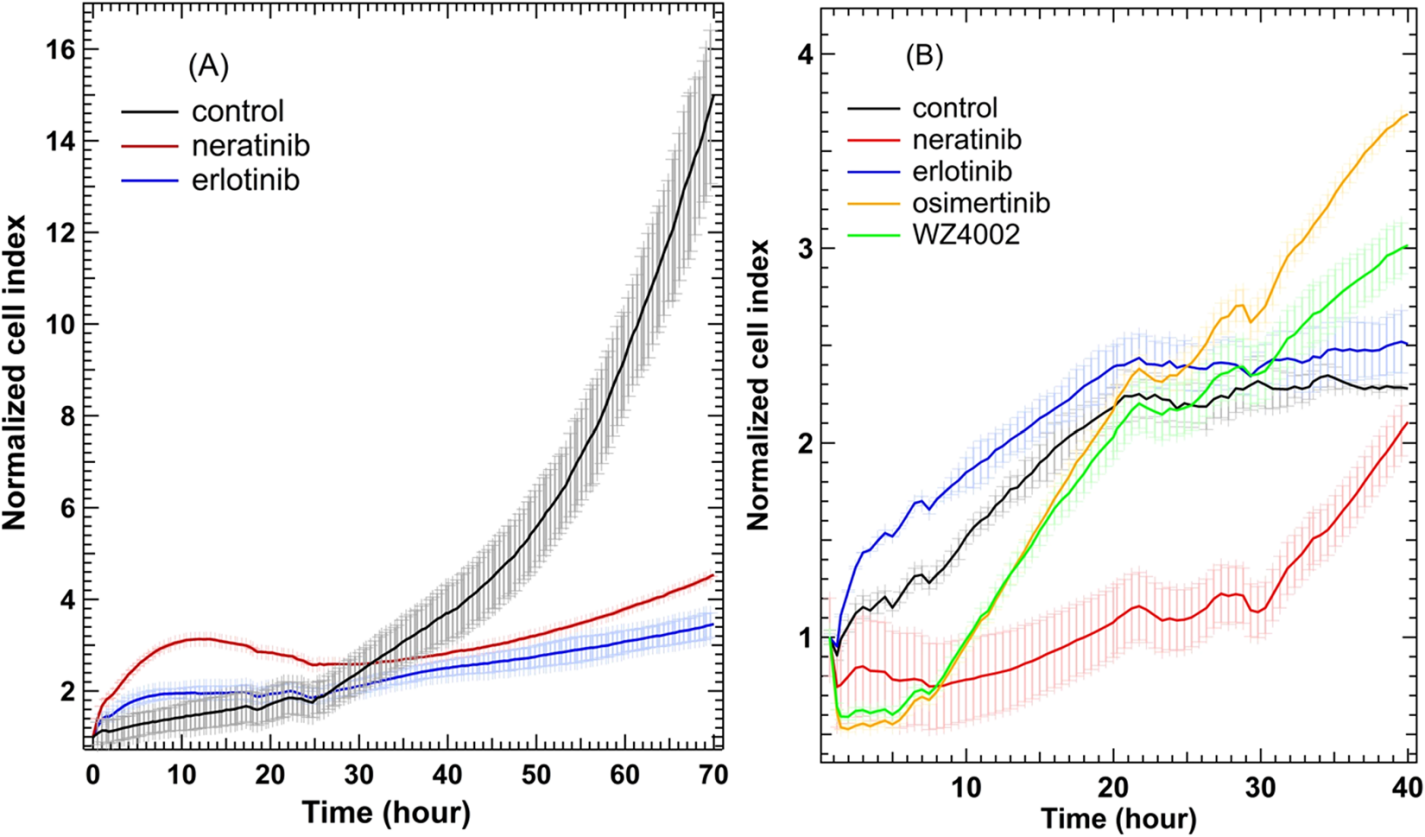
RTCA of Calu-3 (**A**) and NCI-H1975 (**B**) cells of control and cells-treated with erlotinib, neratinib, osimertinib, and WZ4002.

To check that the observed spectral changes in the Raman difference spectra (Figs 1 and 2) are produced as a result of a cellular response to drugs, RTCA method was used. The *xCELLigence* RTCA technology, impedance-based detection of cell viability, has recently been developed as a real-time, non-invasive, and label-free technique to evaluate cellular proliferation, migration and invasion^79^. The kinetics of cell proliferation and cell response to erlotinib, neratinib, osimertinib, and WZ4002 were assessed by the *xCELLigence* platform as shown in Fig. 3. The cell index values of Calu-3 cells (control) increased over time (Panel A) implying that cells still proliferate. The cell index values for cells-treated with neratinib or erlotinib were significantly lower than that of the control after ~35 hours incubation. In case of NCI-H1975 cells treated with neratinib (Panel B), the cell index value is smaller than that of the control over time implying that the rate of cellular proliferation was reduced in the presence of neratinib. On the other hand, the cell index in case of cells treated with erlotinib is similar to that of the control. Thus, NCI-H1975 cells responded to neratinib but showed resistance to erlotinib. In addition, NCI-H1975 cells (Panel B) responded to both osimertinib and WZ4002. This is because at the beginning of the treatment the cell index values of cells treated with these drugs were smaller than those of the control. Moreover, the cell index values became higher than those of the control after ~30  hours of drug treatment. This is may be attributed to the consuming of these drugs after ~30 hours. This is supported by the fact that cells responded to these drugs for a long time when they were treated with higher drug concentrations (25 µM) at which the cell index values were significantly dropped near 0.5–1 even after 20 hours of drug treatment (Fig. S9). Similarly, Raman results showed that NCI-H1975 cells responded to neratinib, WZ4002, and osimertinib but not erlotinib. Therefore, the observed spectral changes in the Raman difference spectra in Figs 1 and 2 can be explained in terms of the integral physiologically relevant cellular responses to TKIs.

**Figure 4:**
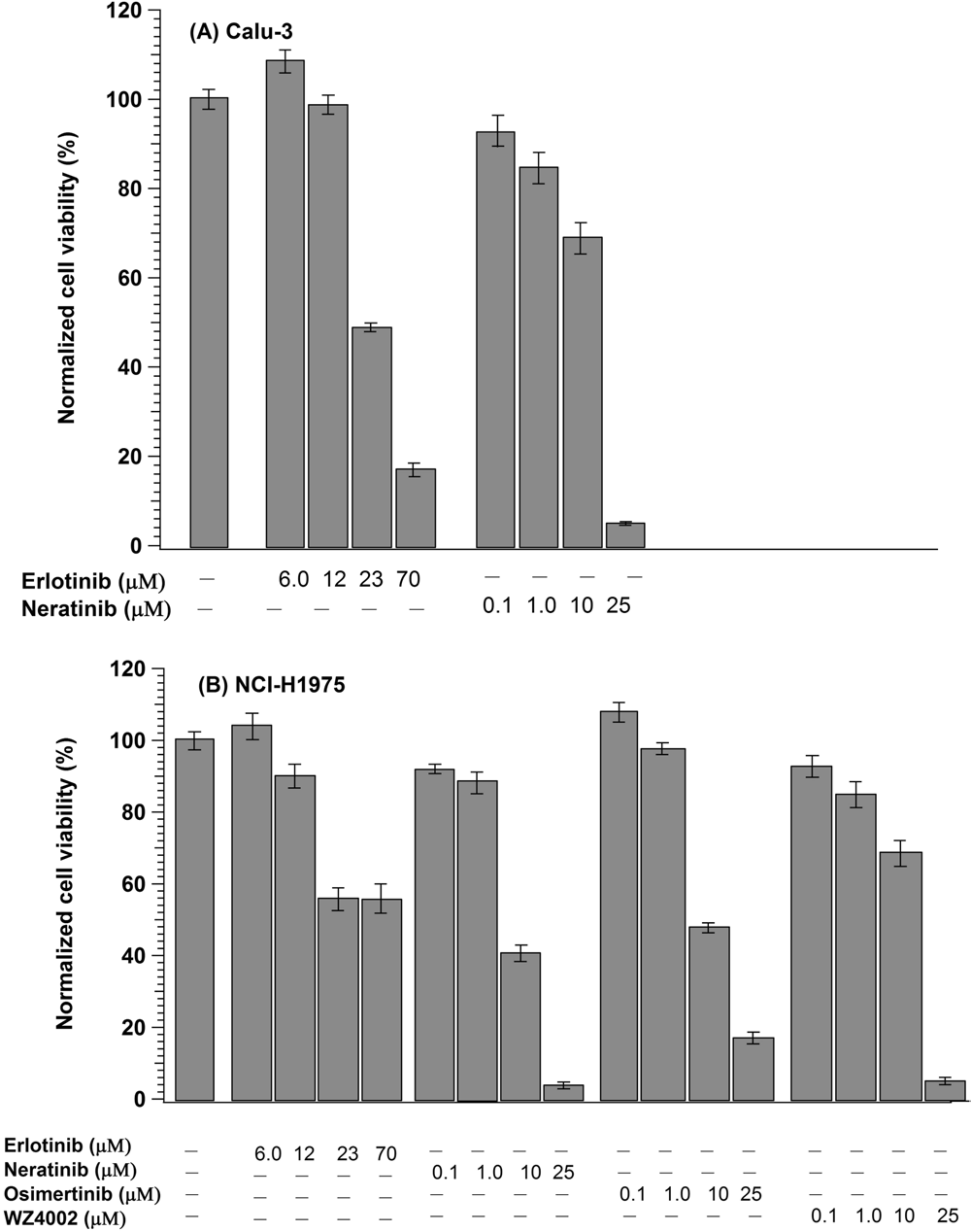
Effect of different TKIs on the viability of (**A**) Calu3 and (**B**) NCI-H1975 cells by MTT. The values are means of four replicates and normalized with respect to the control.

The MTT assay was performed (Fig. 4) and the cell viability decreased as the drug doses increase in both Calu-3 (A) and NCI-H1975 (B) cells. These results are consistent with the RTCA results (Figs 3 and S9) in which the cell index values are significantly dropped as the concentration of TKIs increased. However, the cell viability of erlotinib-treated NCI-H1975 cells was decreased in contrast to the label-free RTCA results that showed resistance to erlotinib (Fig. 3). The reproducibility of the present MTT results for erlotinib-treated NCI-H1975 cells was confirmed. It is noteworthy to mention that the MTT results of erlotinib-treated NCI-H1975 cells are controversial in the literature^80–82^. For instance, the viability of the NCI-H1975 cells was significantly decreased as the erlotinib concentration increases in some studies^80,81^, while it remains constant in another study^82^. It is also reported that the MTT assay is suffering from many limitations such as background absorbance arises from undesired chemical interference and pipetting reproducibility of the replicates^83^.

**Figure 5:**
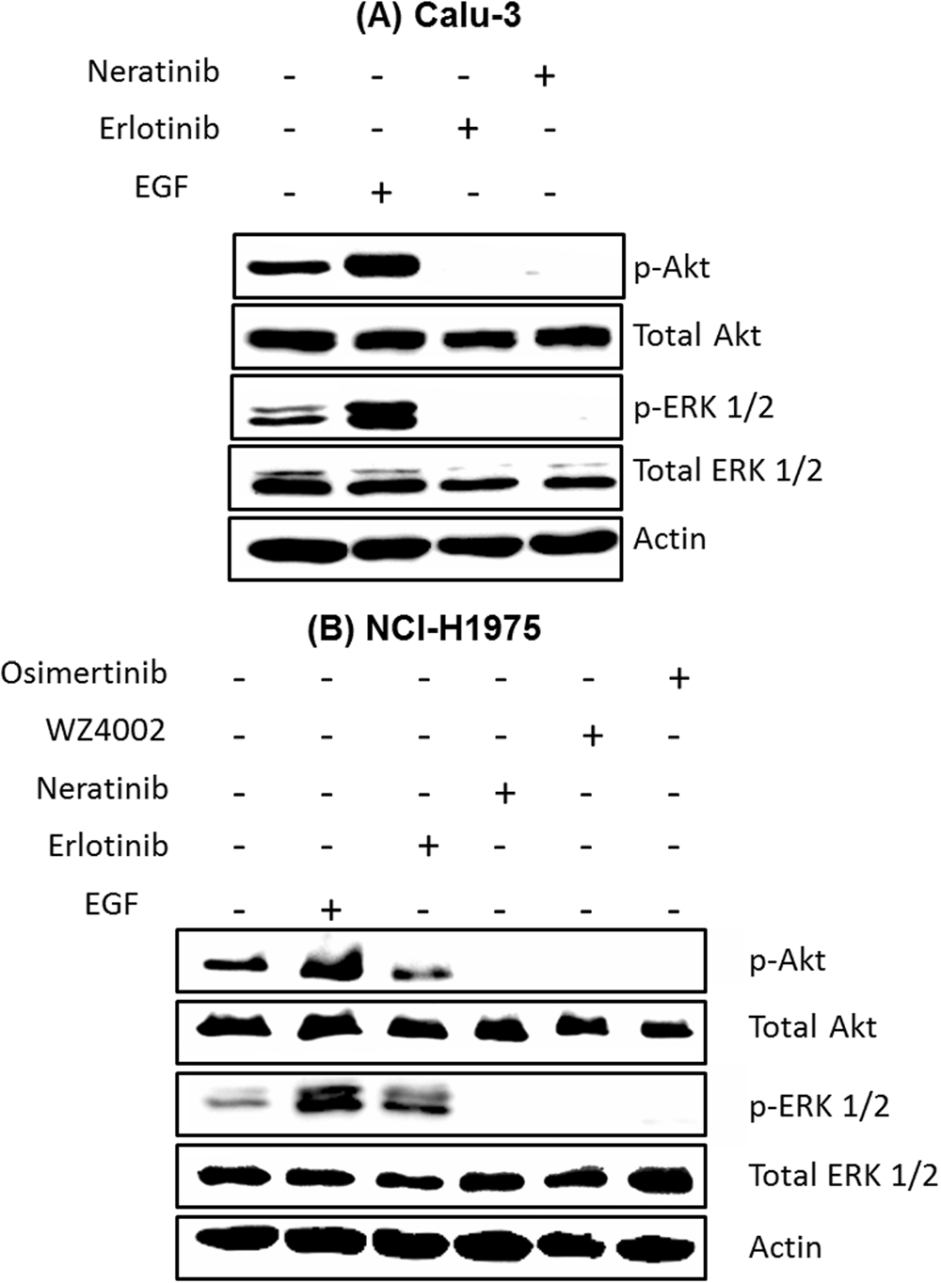
Effect of different TKIs on ERK and AKT phosphorylation in Calu-3 (**A**) and NCI-H1975 (**B**) cells. The lysates of cells were resolved by SDS-PAGE and Western blot analysis using antibodies that recognize phosphospecific ERK1/ERK2 (p-ERK1/2), AKT (p-Akt), total ERK1/ERK2 (ERK1/2), and total Akt. β-actin was used as a loading control.

Western blot is one of the most common *in vitro* methods used to evaluate the potency and efficacy of drug candidates during an early stage of drug discovery. It was used to access the phosphorylation of downstream signalling pathways of EGFR such as extracellular-signal-regulated kinases (ERK1/2) and Protein kinase B (AKT). Calu-3 (Panel A) and NCI-H1975 (Panel B) cells showed ERK and AKT phosphorylation in the absence of drugs (Figs. 5 and S10). This phosphorylation was inhibited in the presence of neratinib in both cell lines compared with that of their corresponding controls. Thus, the observed large spectral changes in Raman difference spectra in Fig. 1 are produced as a result of EGFR inhibition in Calu-3 and NCI-H1975 cells. The Western blot results also indicated that the ERK and AKT phosphorylation was only inhibited in Calu-3 cells treated with erlotinib (Fig. 5A) but it was not inhibited in NCI-H1975 cells (Fig. 5B) as expected due to the resistance of NCI-H1975 cells to erlotinib. Thus, the Western blot results are in agreement with the present RTCA, Raman results, and the clinical observations, where patients with T790M EGFR mutation do not respond to erlotinib^48–51^.

Furthermore, the Western blot results of NCI-H1975 cells indicated that the ERK and AKT phosphorylation was inhibited in the presence of osimertinib or WZ4002 in comparison with that of their corresponding control (Fig. 5B). These results are in agreement with those obtained *in vivo* using mouse lung cancer model harbouring EGFR L858R/T790M, where AKT and ERK1/2 phosphorylation was inhibited by WZ4002, whereas it showed no significant effect on the AKT and ERK1/2 phosphorylation in WT EGFR^57^. Thus, the observed large spectral changes in Raman difference spectrum in Fig. 2 are produced as a result of T790M EGFR inhibition. These results are compatible with the present Raman and RTCA results, *in vivo*, and clinical studies^57,61,76^. The unprocessed full-length blots are presented in Fig. S10.

### Resistance of third-generation TKIs

**Figure 6:**
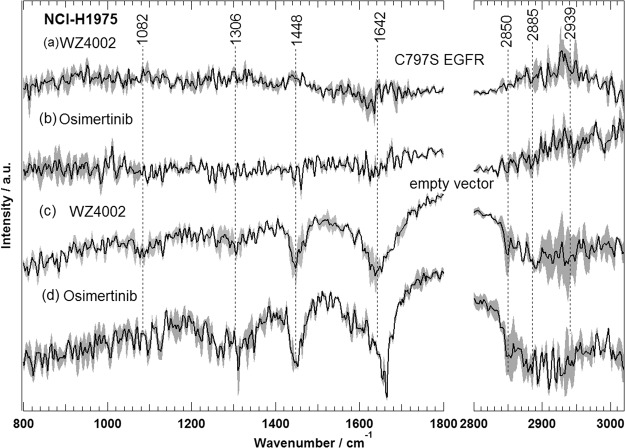
Raman difference spectra of NCI-H1975 cells with (a,b) and without (c,d) C797S EGFR mutation of control — cells treated with either WZ4002 (a,c) or osimertinib (b,d).

Two third of patients with T790M EGFR mutation respond to third-generation EGFR TKIs^57–59^. However, resistance is developed after treatment of patients with these inhibitors due to C797S EGFR mutation^62,63^. To study this acquired resistance by Raman micro-spectroscopy, T790M EGFR cells, erlotinib-resistant cells (NCI-H1975) were made resistant also to third generation TKIs by inducing an additional C797S EGFR mutation in these cells as explained in the experimental section (see SI). The Raman mean spectra of NCI-H1975 cells with and without C797S EGFR mutation in addition to these cells treated with either WZ4002 or osimertinib are displayed in Fig. S11. The Raman difference spectra of cells without drug-treatment versus cells treated with WZ4002 (a,c) and osimertinib (b,d) are displayed in Fig. 6 for NCI-H1975 cells with (a,b) and without C797S EGFR mutation (c,d: empty vector (insertion vector without the mutated receptor gene)). Spectra (a,b) reveal no significant spectral changes, while spectra (c,d) display spectral changes near 2940–2850 (C-H stretching; lipids and proteins), 1642 (amide I; proteins), 1448 (C-H deformation; lipids), 1330–1270 (amide III; proteins and/or nucleic acids), and 1082 cm^−1^ (phospholipid C-C stretching; lipids)^67^. These results clearly show that NCI-H1975 cells with C797S EGFR mutation, do not respond significantly to osimertinib or WZ4002, while those without this mutation responded to either osimertinib or WZ4002. Thus, these results monitored for the first time the acquired resistance to third generation TKIs by Raman micro-spectroscopy at a cellular level. This is in agreement with the observed responses of NSCLC patients with C797S EGFR mutations to TKI therapies^62^.

We also monitored the effect of osimertinib and WZ4002 on NCI-H1975 cells with and without C797S EGFR mutation using the conventional *in vitro* analytical techniques such as RTCA, fluorescence microscopy, and Western blotting. The kinetics of cell proliferation and cell response to osimertinib and WZ4002 were evaluated by the RTCA as shown in Fig. S12. In the case of NCI-H1975/C797S EGFR cells, the signal growth rate for treated cells with osimertinib and WZ4002 is similar to that of the control implying that cells were resistant to these TKIs. On the other hand, the cellular growth rate of cells treated with osimertinib and WZ4002 is different than that of the control in case of NCI-H1975/empty vector cells. For instance, the cell index values of cells treated with these drugs were lower than that of the control in the first 10 hours after drug treatment. Thus, only NCI-H1975/empty vector cells responded to the drugs but cells with C797S EGFR mutation showed a resistance. Therefore, the observed spectral changes by Raman microscopy in Fig. 6 can be explained in terms of an integral physiologically cellular response to drugs. Furthermore, the internalization of osimertinib- or WZ4002- receptor complex in both cells was confirmed by the fluorescence labelling of EGFR following the same experimental conditions of Raman measurements (Fig. S13).

The Western blotting results of NCI-H1975 cells without C797S EGFR mutation (Figs. S14 and S15) indicated that ERK and AKT phosphorylation was inhibited by osimertinib and WZ4002 in comparing with control cells. On the other hand, ERK and AKT activities were not inhibited by osimertinib and WZ4002 in NCI-H1975 cells with C797S EGFR mutation (Figs. S14 and S15). Thus, the Western blot results are compatible with the Raman results, RTCA results, and also with the observed patient responses in case of osimertinib.

### Potential of Raman micro-spectroscopy in cancer therapy evaluation

In the present study, we have shown by Raman micro-spectroscopy that cells with T790M and T790M/C797S EGFR mutations did not respond to first and third generation TKIs, respectively. Raman micro-spectroscopy provides a spectral read out of the overall biochemical composition of the cell. This is a crucial difference from other *in vitro* assays such as Western blot and high throughput screening assays based on fluorescence those monitor individual proteins and single signal transduction pathway. Signal transduction networks are tremendously complicated, and silencing of certain pathways can cause compensation by other pathways^4–7^. Thus, single protein measurements can be deceptive. For instance, we have shown recently that oncogenic K-Ras mutation blocks the response to anti-EGFR therapy using Raman spectral imaging and this is in an agreement with clinical results of patients, but this effect was not observed in the Western blot results^16^. This is most likely due to effects on ERK/AKT phosphorylation triggered by cross-talk and compensation among different signalling pathways that can significantly disturb cellular drug response and in some cases can completely block drug efficacy^84^. On the other hand, the present Raman results are consistent with Western blot results and suggest that cell responses to TKIs are dependent on whether EGFR is mutated (T790M and C797S) or not. These results are in agreement with clinical studies, which show acquired resistance in lung cancer therapy. Therefore, Raman spectral imaging in combination with cluster analysis can be used to evaluate the potency and efficacy of TKIs as well as it has a potential to predict drug resistance.

It is noteworthy to mention that both RTCA and Raman micro-spectroscopy are non-invasive and label-free techniques which can be used to evaluate cell response to drugs. RTCA uses the impedance detection for monitoring various living cell characteristics over time and in the present study, the RTCA results of cellular response to TKIs are compatible with the clinical observations. However, the impedance magnitude is dependent not only on the number of cells but also the cell size and shape, and the cell-substrate attachment quality and may lead to misleading results^85^. In addition, Raman micro-spectroscopy has an advantage in comparison with RTCA, where Raman micro-spectroscopy can provide pharmacokinetic information such as label-free drug distribution and metabolism in cells^31,33–35,37,86^.

Summing up, Raman micro-spectroscopy was used in combination with cluster analysis to explore the acquired drug resistance at a cellular level. The resistance of NSCLC cells to different generations of TKIs was monitored for the first time by label-free Raman micro-spectroscopy. We examined the effect of the first- (erlotinib) and second-generation (neratinib) TKIs, on NCI-H1975 and Calu-3 cells with and without T790M EGFR mutation, respectively. The results indicated that the cellular response to erlotinib was dependent on the T790M EGFR mutation status and cells with T790M EGFR mutation were found to be resistant to erlotinib. Furthermore, cells with T790M EGFR mutation responded to third-generation TKIs such as osimertinib and WZ4002, while those with T790M/C797S EGFR mutations are resistant. Thus, the *in vitro* Raman results indicated that cells with T790M and T790M/C797S mutations are resistant to first- and third-generation TKIs, respectively, which is in agreement with the clinically observed responses of patients. These results show the strength of Raman micro-spectroscopy for detecting the cellular resistance through monitoring the integral cellular responses to different TKIs in comparison with the results of other conventional *in vitro* methods such as fluorescence microscopy, RTCA, MTT assay and Western blotting of single proteins. Therefore, the present study opens a new direction for developing an *in vitro*, simple, non-invasive, and label-free Raman spectroscopic test to decide whether a specific drug can be successfully applied to patients or not.

## Methods

### Cell culture

Human NCI-H1975 (CRL-5908) and Calu-3 (HTB-55) NSCLC cells were obtained from the American Type Culture Collection. Cells were cultured in Dulbecco’s modified Eagle’s medium (DMEM; Invitrogen, Carlsbad, USA) supplemented with 10% fetal bovine serum (FBS; Invitrogen, Carlsbad, USA), 2 mM L-glutamine, and 5% penicillin/streptomycin. Cells were incubated at 37 °C in 10% CO_2_ atmosphere. The cells were sub-cultured regularly when the confluency reached around 80%. The old medium over the cells was discarded from the petri dish and the cells were washed with 10 mL PBS. The cells were detached from the surface of the petri-dish by adding 3 mL of trypsin-EDTA and incubated at 37 °C for 3 minutes. Then the cells were re-suspended in 7 mL medium and centrifuged at 1500 rpm for 3 minutes. Afterwards, the supernatant was discarded and the cell pellet was finally re-suspended in 10 mL medium. From the cell suspension, 3–4 mL was transferred to a new petri-dish, and the volume was completed to 10 mL with DMEM medium. Finally, cells in the petri-dish were checked under a microscope and then incubated at 37 °C in an atmosphere of 10% CO_2_. Once the cell confluences in the petri-dish reached around 80%, the cells were split as described above, and around 15000 cells were added to the CaF_2_ windows (Korth Kristalle, Kiel, Germany) coated with 0.01% Poly-L-lysine and incubated at 37 °C in 10% CO_2_ until the 80% confluence was reached. Cells were treated with erlotinib (23 µM), neratinib (1 µM), osimertinib (1 µM), or WZ4002 (1 µM) at 37 °C in 10% CO_2_ atmosphere for 16 hours to monitor cell response to a drug. These drug concentrations are very close to the detected drug concentrations in the plasma of human patients after treatment with single oral doses^87–92^. Cells were also treated with growth factor (EGF, 50 ng/mL) at 37 °C in 10% CO_2_ atmosphere for 30 minutes. Finally, cells were fixed with 4% paraformaldehyde (VWR International, Darmstadt, Germany) for 10 minutes and washed three times by phosphate-buffered saline (PBS, Invitrogen, Carlsbad, USA) buffer. Then the slides were submerged in PBS buffer and stored at 4 °C until used.

### Confocal Raman microscopy

A WITec alpha 300AR confocal Raman microscope (Ulm, Germany) was used to acquire Raman micro-spectroscopic imaging of cancer cells as described previously^16,20,31^. A 532 nm excitation source from a frequency-doubled Nd:YAG laser (Crystal Laser, Reno, USA) was used with the output power of ~40 mW. The laser light was coupled to a Zeiss microscope by using a wavelength-specific single-mode optical fiber. The laser light was collimated and afterwards focused on the sample through a Nikon NIR APO (60×/1.00 NA) water-immersion objective. The sample was placed on a piezoelectrically driven microscope scanning stage. Raman backscattered light was collected by the same water immersion objective and detected by a back-illuminated deep-depletion charge coupled device (CCD) camera cooled at −60 °C. Raman micro-spectroscopic measurements were performed by raster-scanning the laser light over cancer cells to acquire a Raman spectrum at a speed of 0.5 seconds per pixel with a pixel resolution was 500 nm.

## Electronic supplementary material

Supplementary Information
