# Supplementary material for: Raman micro-spectroscopy monitors acquired resistance to targeted cancer therapy at the cellular level
Source: Sci Rep. 2018 Oct 15;8:15278. doi: 10.1038/s41598-018-33682-7 (PMC6189084; doi:10.1038/s41598-018-33682-7)
Supplement: Supplementary file 1 — Supplementary Information [file 41598_2018_33682_MOESM1_ESM.pdf]

Supplementary Information

Raman micro-spectroscopy monitors acquired resistance to targeted cancer therapy at the cellular level

Mohamad K. Hammoud,[1,§] Hesham K. Yosef,[1,§] Tatjana Lechtonen,[1,§] Karim Aljakouch,[1,§] Martin Schuler,[1] Wissam Alsaidi,[1] Ibrahim Daho,[1] Abdelouahid Maghnouj,[2] Stephan Hahn,[2] Samir F. El-Mashtoly,[1,*] and Klaus Gerwert[1]

[1]Department of Biophysics, Ruhr-University Bochum, 44780 Bochum, Germany

[2]Department of Molecular GI-Oncology, Clinical Research Center, Ruhr-University Bochum, 44780 Bochum, Germany

*samir@bph.rub.de

[§]These authors contributed equally to this work

Plasmid construct. A plasmid construct encoding mutated EGFR was PCR-amplified from pBabe EGFR Del1-T790M (addgene; Plasmid # 32072). The following PCR primers were used, forward 5'-CGC TAC CGG TGC CAC CAT GCG ACC CTC CGG GAC G-3', reverse 5'-TCG ACA ATT GTC ATG CTC CAA TAA ATT CAC TG-3' for EGFR Del1-T790M amplification. The PCR product was inserted into AgeI and EcoRI sites of the lentiviral vector pLJM1-EGFP (Addgene, Cambridge, USA; Plasmid #19319), replacing the EGFR cassette. The mutation EGFR Del1-T790M-C797S was engineered into the pLJM1-EGFP Del1-T790M construct using QuickChange II XL Site-Directed Mutagenesis Kit (Agilent Technologies, Waldbronn, Germany). Primer sequences for site-directed mutagenesis were following, sense 5'-CAT GCC CTT CGG CTC CCT CCT GGA CTA-3' and antisense 5'-TAG TCC AGG AGG GAG CCG AAG GGC ATG-3' for EGFR del1-T790M-C797S. The correct insert sequence of the newly generated vector was confirmed by cycle sequencing analysis.

Lentivirus production and lentiviral infection. Lentiviruses were produced by transfecting packaging cells (HEK293T) with a 3-plasmid system. For transfections 12 μg pCMVΔR8.2 (Addgene, Cambridge, USA; Plasmid # 12263), 6 μg pHIT G (Addgene, Cambridge, USA; Plasmid #8454) and 12 μg pLJM1 plasmid DNA were combined with 62 μl of 2 M $CaCl_2$ in a final volume of 500 μl. Subsequently, 500 μl of 2x HBS phosphate buffer was dropwise added to the mixture and incubated for 10 min at room temperature. The 1 ml transfection mixture was then added to 50% confluent HEK293T-cells (seeded the day before) into a 10 cm plate. Cells were incubated for 16 h (37°C and 10% CO_2), and the medium was changed to remove remaining transfection reagent. 36 h post transfection lentiviral-supernatants were collected and filtered (0.45 μm pore size) for subsequent infection of target cells. 10 ml supernatant containing 4 μg/ml polybrene was immediately used to infect target cells seeded the day before in 10 cm plates to reach 50% confluency on

the day of infection. Cells were incubated for 24 h, and then the medium was changed to remove virus particles. To control infection rate, a parallel infection under identical conditions and targeting the same cell line was prepared using a lentiviral-GFP expression control vector pRRLU6-CPPT-pSK-GFP (kindly provided by Sheila Stewart).

Immunofluorescence staining and fluorescence microscopy. After Raman measurements of cells grown on CaF_2 windows, the formalin-fixed cells were permeabilized with 0.2% Triton X-100 for 5 min at room temperature, washed with PBS, and blocked with 1% bovine serum albumin (BSA) for 30 min. Cells were incubated with the primary rabbit monoclonal anti-EGFR (ab30; Abcam, Cambridge, UK) and primary mouse monoclonal anti-HER2 (ab8054); Abcam, Cambridge, UK) overnight at 4°C, followed by washing with PBS buffer and incubation for 1 h at room temperature with fluorescein- (FITC; Jackson ImmunoResearch, West Grove, USA) and rhodamine (TRITC; Jackson ImmunoResearch, West Grove, USA) conjugated secondary antibodies, respectively. Excess antibodies were removed by washing with PBS buffer several times. Cells were additionally incubated with 1,5-bis{[2-(dimethylamino)ethyl]amino}-4,8 dihydroxyanthracene-9,10-dione (DRAQ-5; Cell Signaling Technology, Danvers, USA) for 15 min. Finally, cells were washed with PBS buffer.

Fluorescence imaging was acquired with a confocal microscope (Leica TCS SP5 II) using a Leica HCX PL APO (25×/0.95 NA) water-immersion objective. The fluorescence of nucleus was imaged by exciting with the 633 nm laser, whereas that of HER2 and EGFR was imaged using the 488 and 561 nm excitation lasers, respectively.

Real-time cell analysis (RTCA). *In vitro* RTCA assay was performed using the xCELLigence DP system (Bioscience, Germany). Suspension of cells was diluted to final concentration of 10000 cells/100 µl in each well of 16-well E-plates and was incubated at 37°C for 24 hours. Then 50 µl of the medium was removed from each well, and 50 µl of each

of erlotinib (23 µM), neratinib (1 µM), osimertinib (1 µM), and WZ4002 (1 µM) were added to the wells. The proliferation of NCI-H1975 and Calu-3 cells was monitored by xCELLigence system software. The quantification of cell proliferation was determined by the cell index value based on the detected cell-electrode impedance in each well. The cell index values were normalized using cell index value at the time point of adding drugs to cells and acquired every 15 minutes for around 140 hours.

Cell viability assay. To determine the effect of TKIs on cell growth, cell viability was detected using 3-(4,5-dimethylthiazol-2-yl)-2,5- diphenyl tetrazolium bromide (MTT). MTT (yellow) is converted by viable cells into violet formazan by mitochondrial reductases, and thus it is used as an indicator of the cell viability. Cells were seeded to a density of 5 x 10^3 cells/well in 96-well plates and incubated for 24 hours. Calu-3 and NCI-H1975 cells were incubated with erlotinib (6.0, 12, 23, and 70 µM) and neratinib (0.1, 1.0, 10, and 25 µM) for 16 hours. NCI-H1975 cells were also incubated with osimertinib and WZ4002 (0.1, 1.0, 10, and 25 µM) for 16 hours and 100 µl of each drug was added to each well.

A volume of 50 µl of MTT (2.5 mg/ml) was then added to each well and the plates were incubated for 2 hours at 37°C in a 10% CO_2 humidified incubator. After this incubation period, the medium was discarded and 200 µl of Dimethylsulfoxide (DMSO) was added to each well to extract the dye. The plates were shaken for 15 min at 240 times/minute and absorbance was measured at 550 nm with a reference filter of 620 nm using a microplate reader (TECAN Sunrise, Austria). Four replicate wells were used for each concentration. Cell viability in each sample was normalized to that of the control sample.

Western blot analysis. A buffer containing phosphatase inhibitor mixture II (Sigma-Aldrich, München, Germany) and protease inhibitor cocktail (Roche, Mannheim, Germany) was used to harvest cells. Cells were resolved by SDS-PAGE using 10% polyacrylamide gels and transferred to Immobilon-P (Millipore, Hessen, Germany) membranes. After that, the

membranes were incubated with antibodies to phosphorylated ERK1 and ERK2 (p ERK1/2; Cell Signaling Technology, Danvers, MA, USA), total ERK1 and ERK2 (Total ERK1/2; Cell Signaling Technology, Danvers, MA, USA), phosphorylated AKT (p-AKT; Cell Signaling Technology, Danvers, USA), total AKT (Cell Signaling Technology, Danvers, USA), and β-actin (Cell Signaling Technology, Danvers, USA). The antibodies were identified with the appropriate anti-mouse and anti-rabbit horseradish peroxidase-conjugated secondary antibody enhanced by chemiluminescence (Pierce, Life Technologies GmbH, Darmstadt, Germany). Images were taken with the Versa Doc 5000 imaging system (Bio-Rad, München, Germany).

Multivariate analysis. Raman hyperspectral data were imported into Matlab 8.2 (The MathWorks, Natick, MA, USA). In-house built scripts were used for data preprocessing and multivariate analyses. The spectra of background (PBS buffer), which lack a C–H band at 2800–3000 cm^{-1} were deleted. An impulse noise filter was applied to remove cosmic spikes and the Raman spectra were interpolated to a reference wavenumber scale. After that Raman spectra were baseline-corrected by using a third-order polynomial. In addition, the spectra were vector normalized. HCA was calculated on the regions of 700–1800 and 2800–3050 cm^{-1} using Ward's clustering combined with Pearson's correlation distance.

To obtain Raman average spectra of both the control and cells-treated drug, the different clusters of the HCA of each Raman measurement were merged together to produce one mean spectrum of each measurement. Thereafter, all mean spectra of all measurements of control and cells-treated drug were averaged to produce Raman average spectra for control and cells-treated drug. Finally, the Raman difference spectra of cells (control — cells-treated drug) were calculated using normalized average spectra using the phenylalanine band near 1006 cm^{-1}. The Raman average spectra of cellular components such as membrane, cytoplasm, nucleus, and lipid droplets were calculated as described previously.[1]

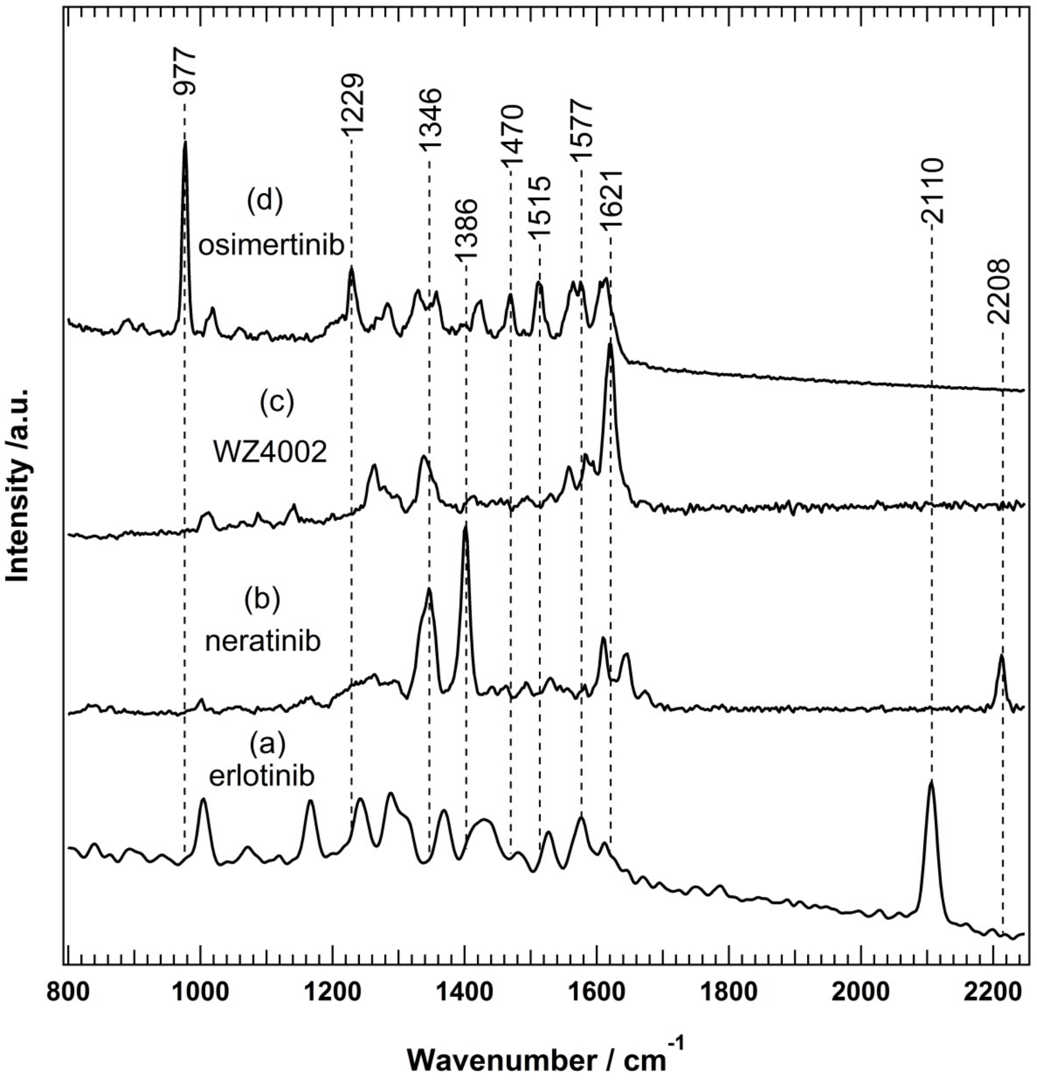

Figure S1. (A) The Raman spectra erlotinib (a), neratinib (b), WZ4002 (c), and osimertinib (d).

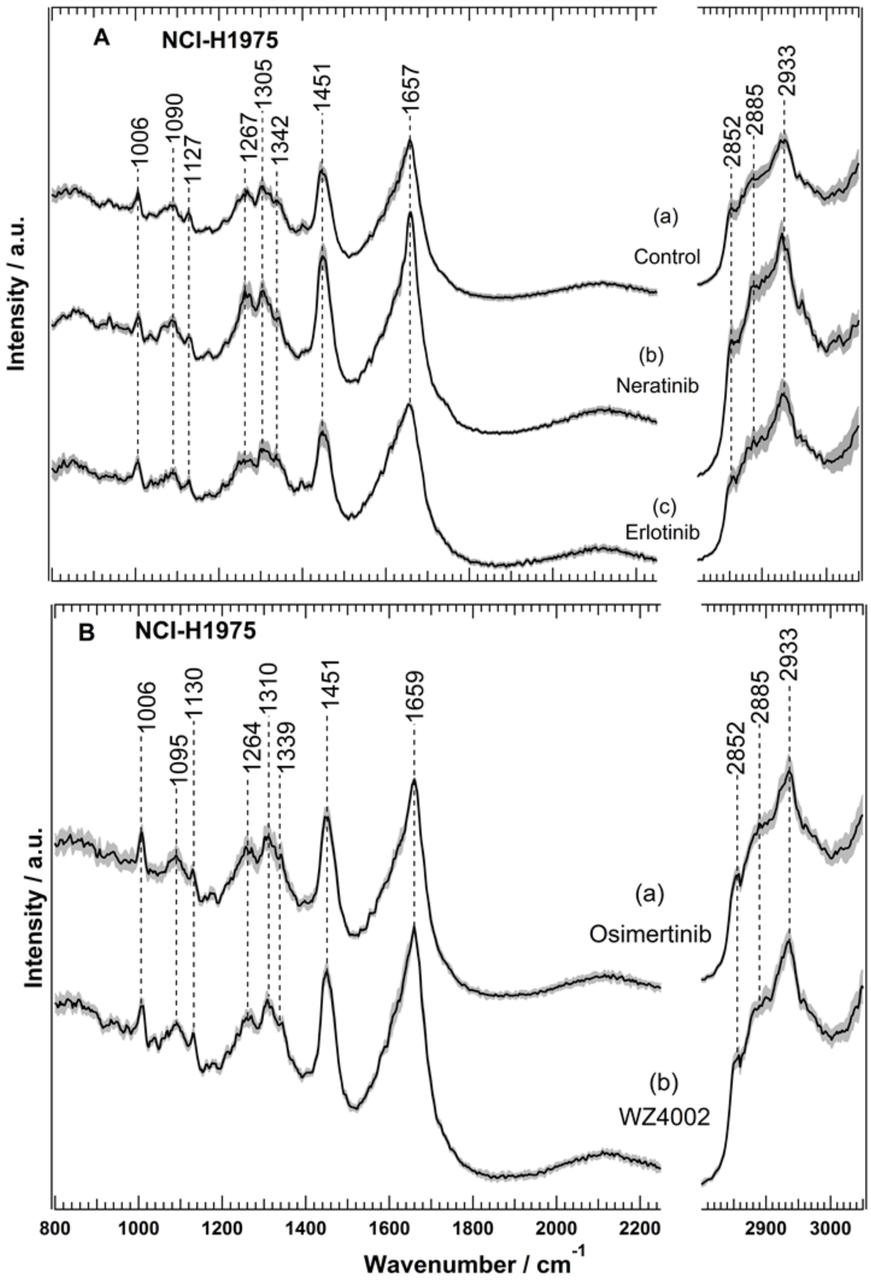

Figure S2. (A) The average cluster Raman spectra of NCI-H1975 cells control (a), neratinib-treated cells (b), and erlotinib-treated cells (c) in the 750−1800 and 2800−3020 cm^{-1} regions. (B) The average cluster Raman spectra of osimertinib-treated cells (a) and WZ4002-treated cells (b) are also shown. Shading represents the standard deviation.

It is obvious that the average spectra of drug-treated cells (Figure S2) are free from the contribution of drug bands (Figure S1). For instance, the strong erlotinib (2110 cm^{-1}), neratinib (2208 and 1386 cm^{-1}), WZ4002 (1621 cm^{-1}), and osimertinib (977 cm^{-1}) bands are absent in the corresponding spectra of the drug-treated cell. Therefore, the observed spectral changes in the cell upon drug treatment are free from drug contribution.

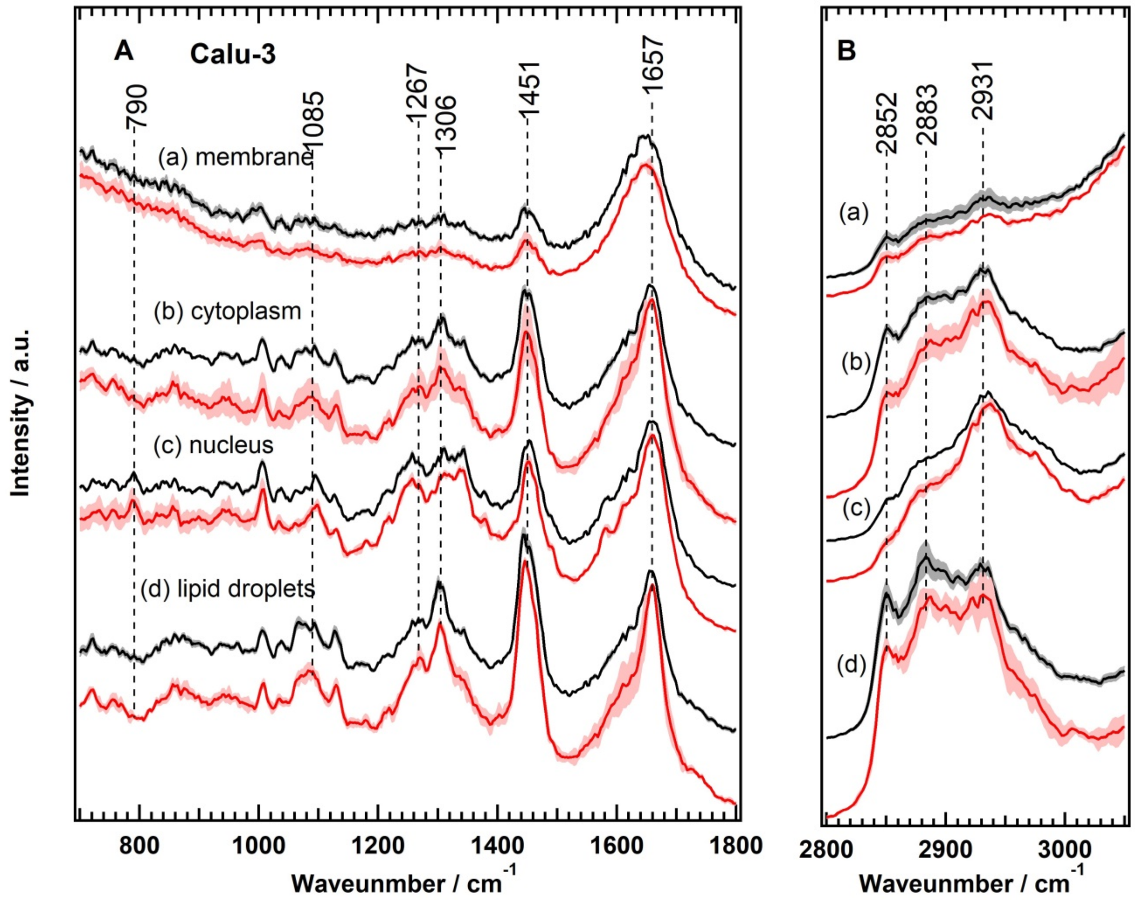

Figure S3. The average cluster Raman spectra of the plasma membrane (a), cytoplasm (b), nucleus (c), and lipid droplets (d) in the 750−1800 and 2800−3020 cm^{-1} regions. The spectra of control and neratinib-treated Calu-3 cells are shown in black and red, respectively. Shading represents the standard deviation.

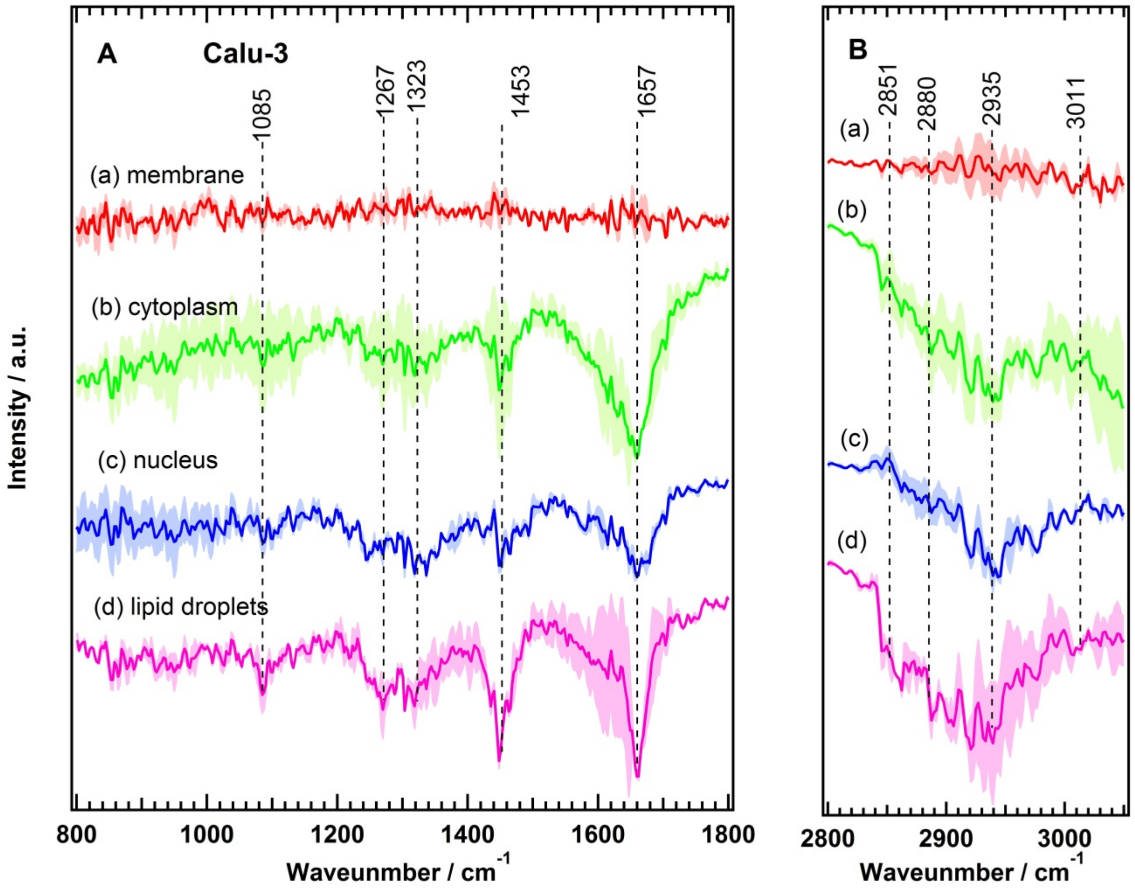

Figure S4. Effect of neratinib on Calu-3 subcellular components. Raman difference spectra of control cells versus cells exposed to 1 μM neratinib for the plasma membrane (a), cytoplasm (b), nucleus (c), and lipid droplets (d). Shading represents the standard deviation.

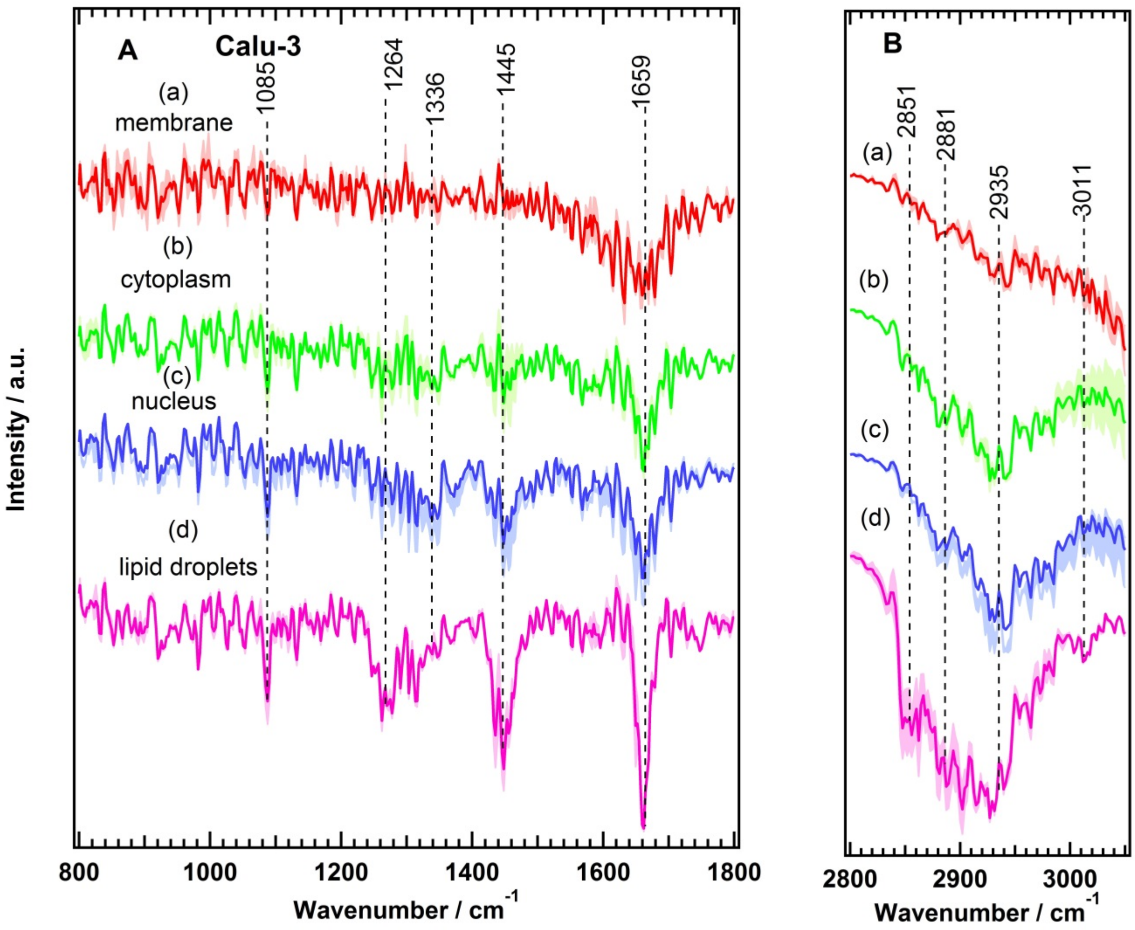

Figure S5. Effect of erlotinib on Calu-3 subcellular components. Raman difference spectra of control cells versus cells exposed to 23 µM erlotinib for the plasma membrane (a), cytoplasm (b), nucleus (c), and lipid droplets (d). Shading represents the standard deviation.

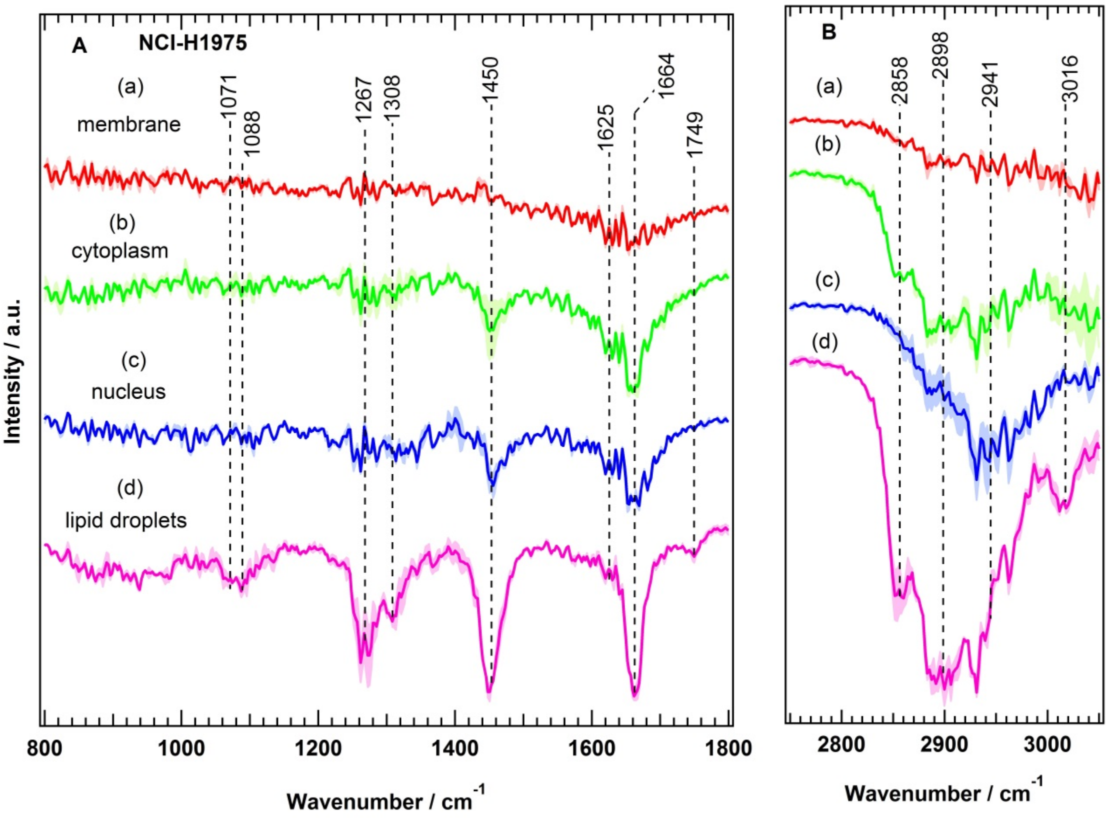

Figure S6. Effect of neratinib on NCI-H1975 subcellular components. Raman difference spectra of control cells versus cells exposed to 1 μM neratinib for the plasma membrane (a), cytoplasm (b), nucleus (c), and lipid droplets (d). Shading represents the standard deviation.

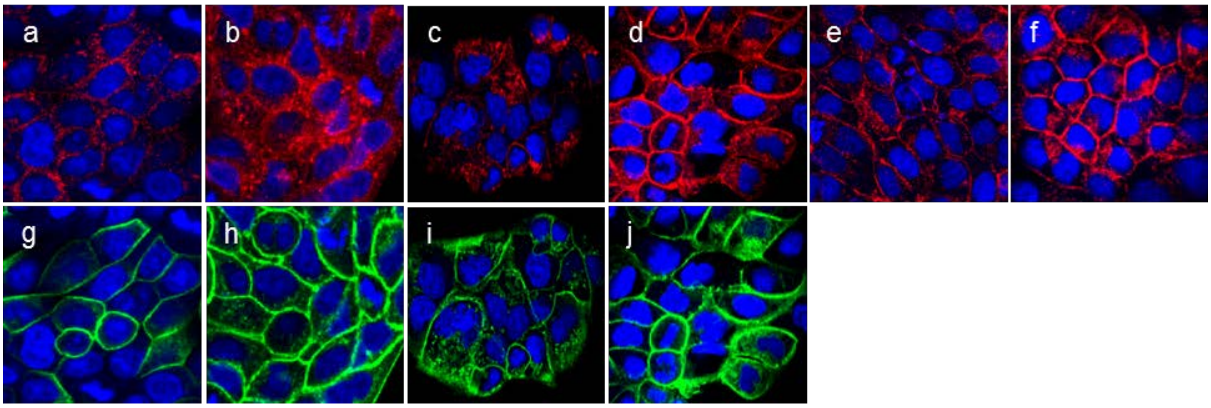

Figure S7. Fluorescence imaging of Calu-3 cells: control (a,g), cells treated with either EGF (b,h), erlotinib (c,i), neratinib (d,j), WZ4002 (e), and osimertinib (f). The nucleus, EGFR, and HER2 are shown in blue, red and green, respectively.

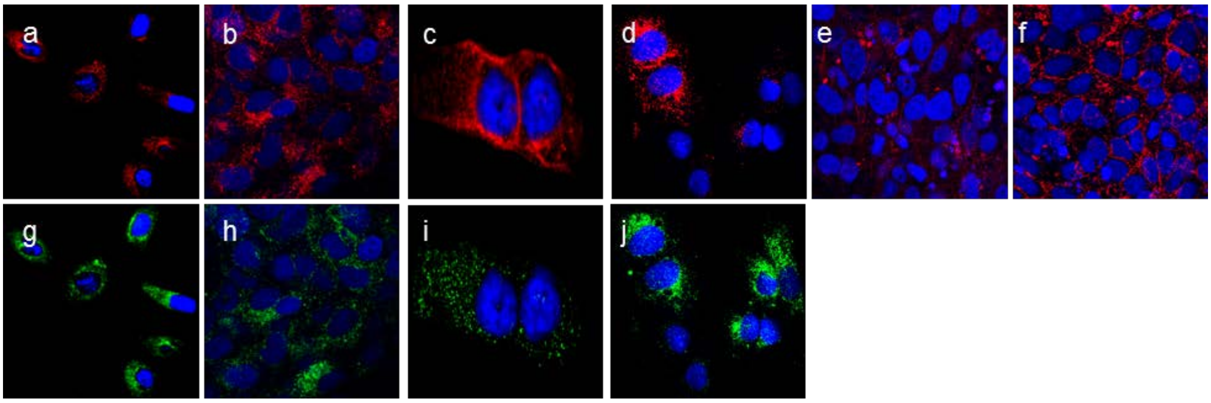

Figure S8. Fluorescence imaging of NCI-H1975 cells: control (a,g), cells treated with either EGF (b,h), erlotinib (c,i), neratinib (d,j), WZ4002 (e), and osimertinib (f). The nucleus, EGFR, and HER2 are shown in blue, red and green, respectively.

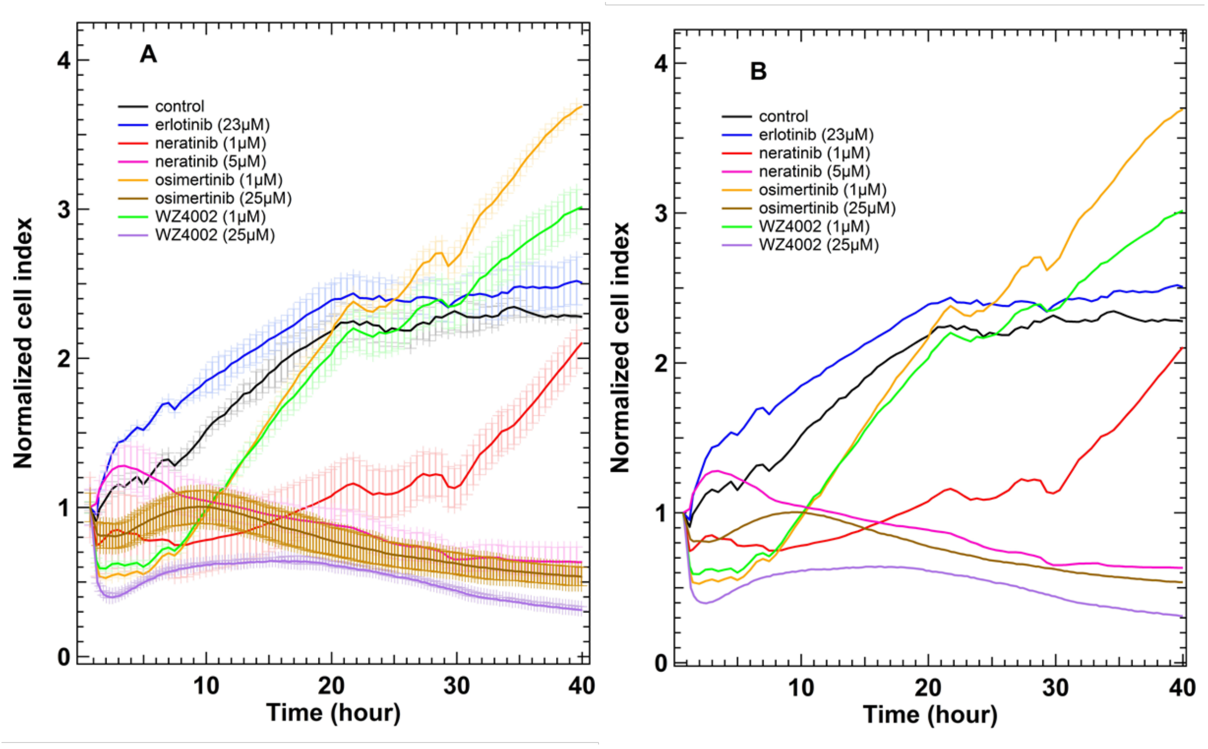

Figure S9. RTCA of NCI-H1975 cells of control and cells treated with erlotinib, neratinib, osimertinib, or WZ4002 with (A) and without (B) the standard deviation.

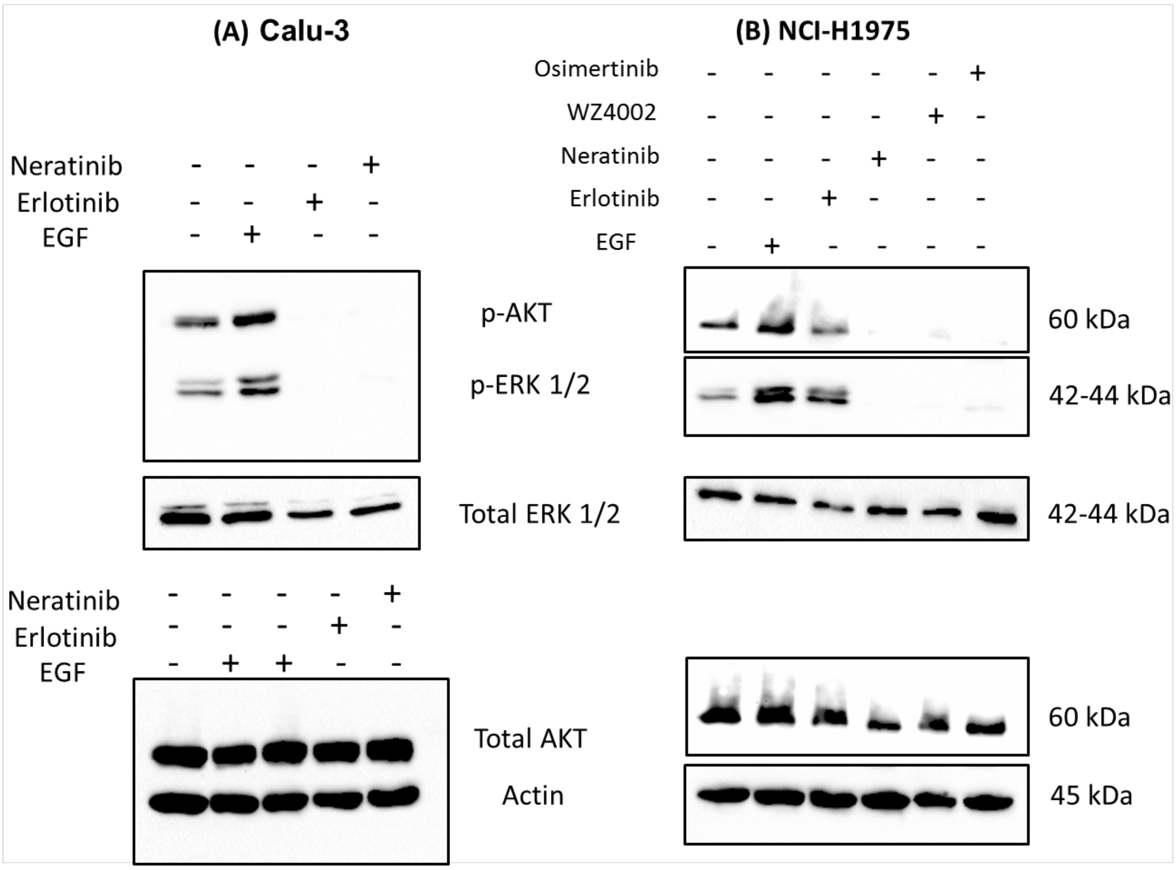

Figure S10. The unprocessed full-length blots those shown in Figure 5.

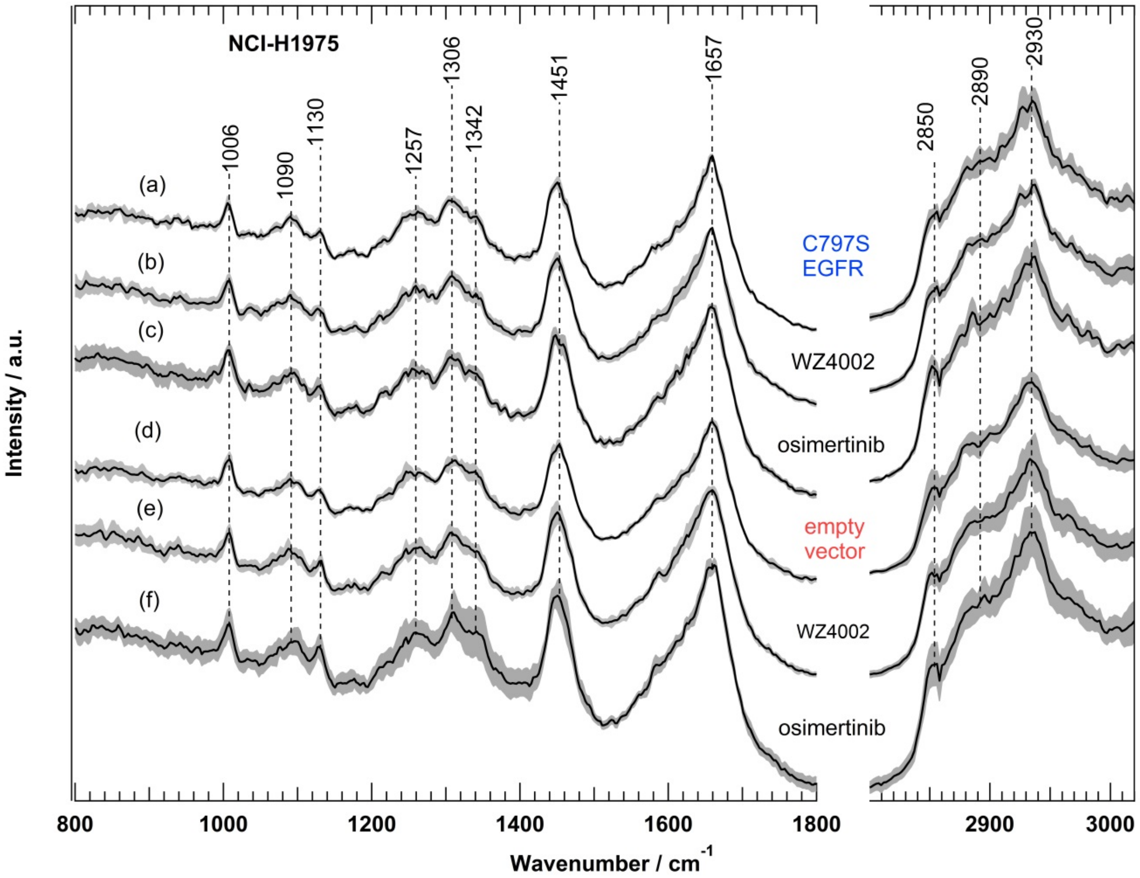

Figure S11. Raman average spectra of NCI-H1975 cells with (a-c) and without C797S EGFR (d-f) mutation. The spectra represent cells with WZ4002 (b,e) or osimertinib (c,f) treatment or without treatment (a,d).

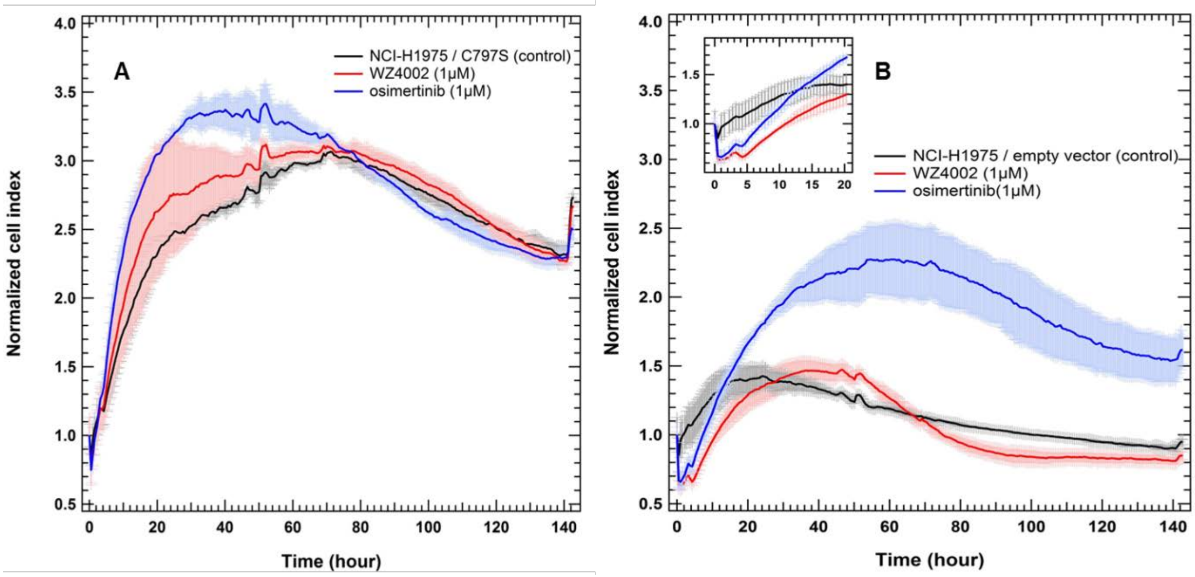

Figure S12. RTCA of NCI-H1975 cells with (A) and without C797S EGFR (B) mutation. Cells of control and cells-treated osimertinib or WZ4002 are shown. An inset of Panel B shows the effect of drugs in the first 20 hours after drug treatment.

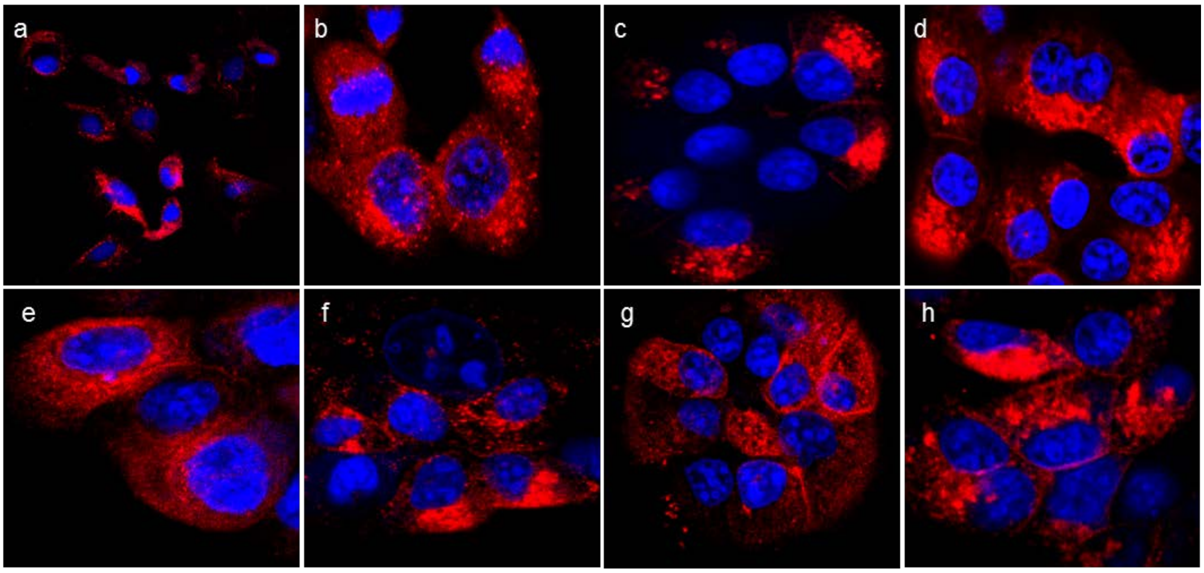

Figure S13. Fluorescence imaging of NCI-H1975 cells with (a-d) and without (e-h) C797S EGFR mutation: control (a,e), cells treated with either EGF (b,f), osimertinib (c,g) and WZ4002 (d,h). The nucleus and EGFR are shown in blue and red, respectively.

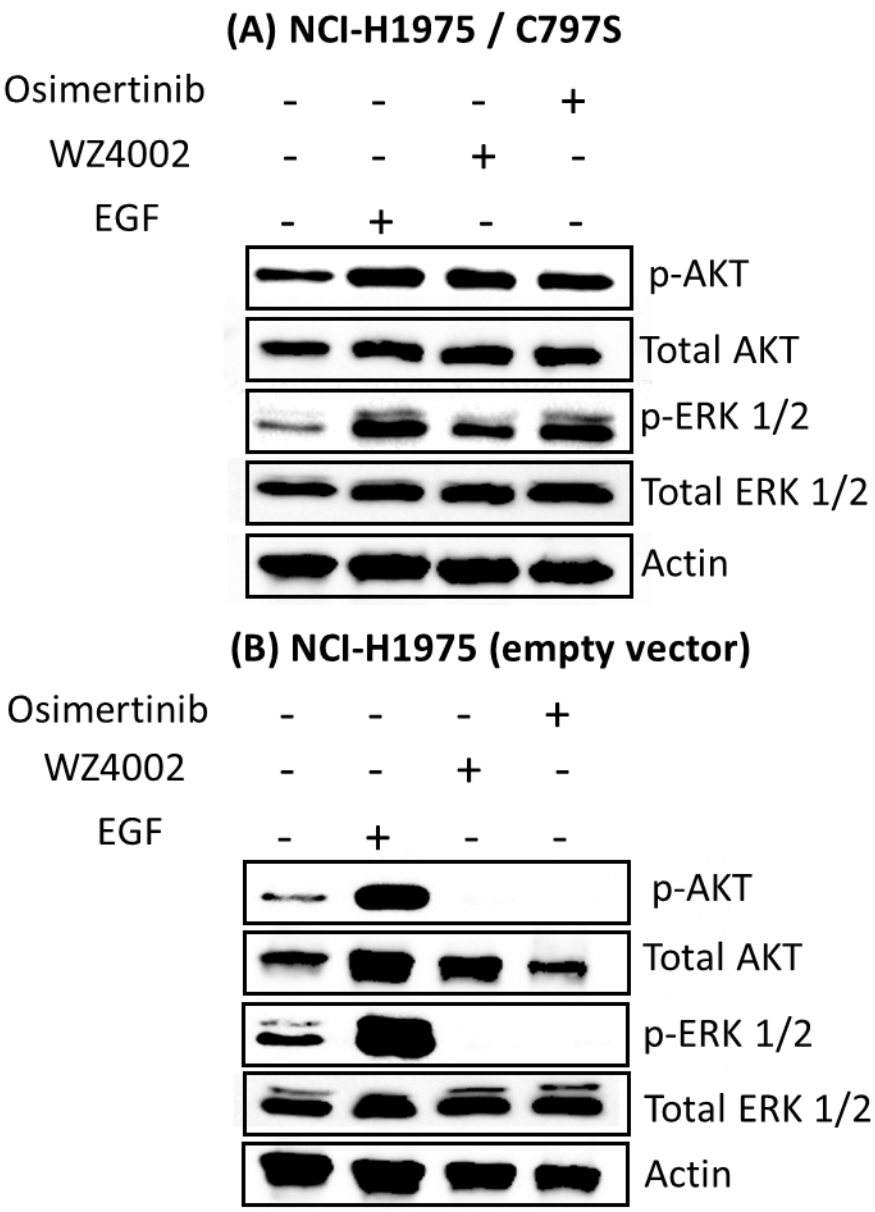

Figure S14. Effect of different TKIs on ERK and AKT phosphorylation in NCI-H1975 cells with (A) and without (B) C797S EGFR mutation. The lysates of cells were resolved by SDS-PAGE and Western blot analysis using antibodies that recognize phosphospecific ERK1/ERK2 (p-ERK1/2), AKT (p-AKT), total ERK1/ERK2 (ERK1/2), and total AKT. β-actin was used as a loading control. The full-length blots are shown in Figure S15.

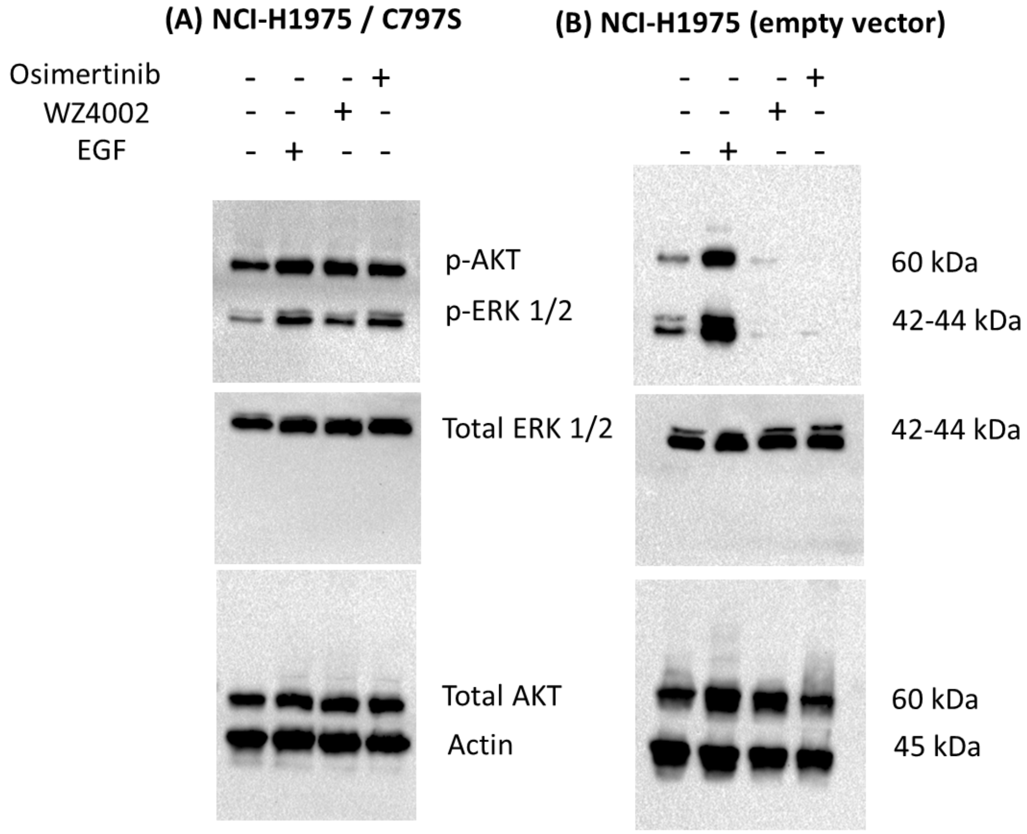

Figure S15. The unprocessed full-length blots those shown in Figure S14.

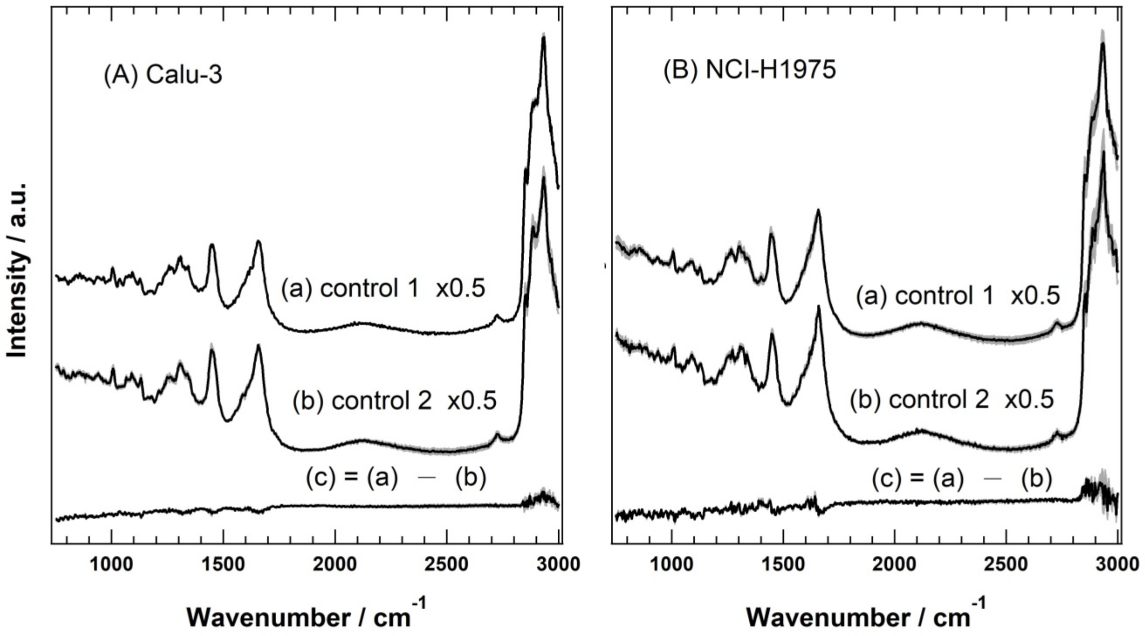

Figure S16. The average cluster Raman spectra of two replicates (a-b) of (A) Calu-3 and (B) NCI-H1975 cells. Shading represents the standard deviation. The difference spectra between different controls (d) of the same cell type display very minor spectral changes.

To confirm that different cellular phases have no significant effect on the observed spectral changes in Figures 1 and 2, we performed Raman measurements of two replicates of control of each cell-line (Calu-3 and NCI-H1975) and approximately 60 cells were measured for each replicate. The average spectrum for each control was calculated, and then the Raman difference spectrum between the two replicates was created as shown in Figure S16. It reveals very minor spectral changes compared with those observed in Figures 1 and 2.

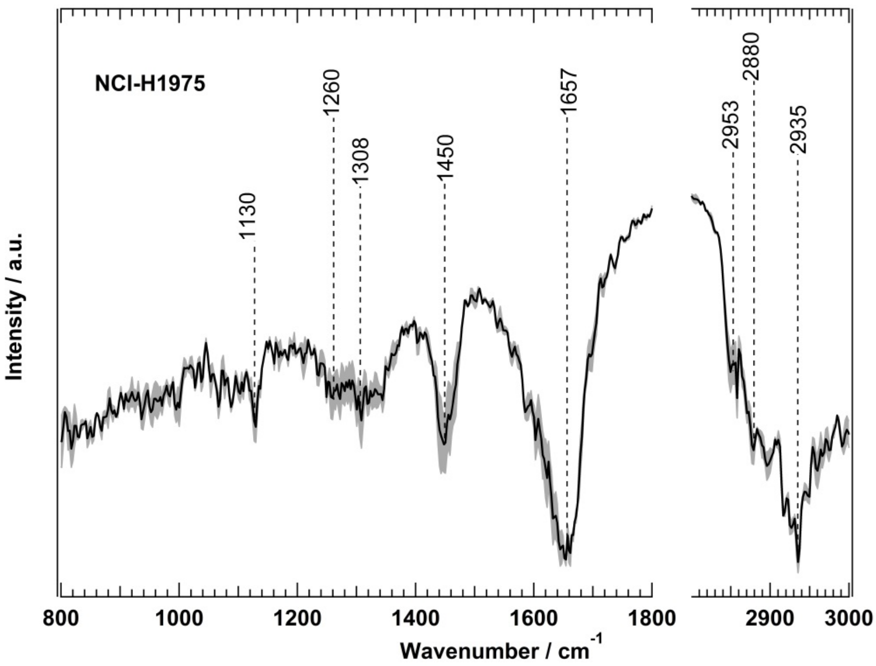

Figure S17. The Raman difference spectrum between NCI-H1975 cells (control) and neratinib-treated NCI-H1975 cells. Shading represents the standard deviation. In these experiments, cells were grown under serum starvation condition[2] to synchronize the cells into G0/G1 phase using DMEM supplemented with 0.1 % fetal bovine serum, 2 mM L-glutamine, and 5% penicillin/streptomycin. Cells were incubated at 37°C in 10% CO_2 atmosphere.

Large spectral changes were observed as shown in Figure S17 similar to those detected for cells grown without serum starvation conditions (Figure 1B(b)). Therefore, different cellular phases if present have a minor contribution to the observed spectral changes (Figures 1 and 2) those mainly produced as a result of drug effect.

References

(1) El-Mashtoly, S. F.; Yosef, H. K.; Petersen, D.; Mavarani, L.; Maghnouj, A.; Hahn, S.; Kötting, C.; Gerwert, K. Label-Free Raman Spectroscopic Imaging Monitors the Integral Physiologically Relevant Drug Responses in Cancer Cells. *Anal. Chem.* **2015**, *87* (14), 7297–7304.

(2) Khammanit, R.; Chantakru, S.; Kitiyanant, Y.; Saikhun, J. Effect of Serum Starvation and Chemical Inhibitors on Cell Cycle Synchronization of Canine Dermal Fibroblasts. *Theriogenology* **2008**, *70* (1), 27–34.
